# Sustained Autophagy Contributes to Measles Virus Infectivity

**DOI:** 10.1371/journal.ppat.1003599

**Published:** 2013-09-26

**Authors:** Clémence Richetta, Isabel P. Grégoire, Pauline Verlhac, Olga Azocar, Joël Baguet, Monique Flacher, Frédéric Tangy, Chantal Rabourdin-Combe, Mathias Faure

**Affiliations:** 1 CIRI, International Center for Infectiology Research, Université de Lyon, Lyon, France; 2 Inserm, U1111, Lyon, France; 3 Ecole Normale Supérieure de Lyon, Lyon, France; 4 Université Lyon 1, Centre International de Recherche en Infectiologie, Lyon, France; 5 CNRS, UMR5308, Lyon, France; 6 Unité de Génomique Virale et Vaccination, Institut Pasteur, CNRS URA-3015, Paris, France; University of New Mexico, United States of America

## Abstract

The interplay between autophagy and intracellular pathogens is intricate as autophagy is an essential cellular response to fight against infections, whereas numerous microbes have developed strategies to escape this process or even exploit it to their own benefit. The fine tuned timing and/or selective molecular pathways involved in the induction of autophagy upon infections could be the cornerstone allowing cells to either control intracellular pathogens, or be invaded by them. We report here that measles virus infection induces successive autophagy signallings in permissive cells, *via* distinct and uncoupled molecular pathways. Immediately upon infection, attenuated measles virus induces a first transient wave of autophagy, *via* a pathway involving its cellular receptor CD46 and the scaffold protein GOPC. Soon after infection, a new autophagy signalling is initiated which requires viral replication and the expression of the non-structural measles virus protein C. Strikingly, this second autophagy signalling can be sustained overtime within infected cells, independently of the expression of C, but *via* a third autophagy input resulting from cell-cell fusion and the formation of syncytia. Whereas this sustained autophagy signalling leads to the autophagy degradation of cellular contents, viral proteins escape from degradation. Furthermore, this autophagy flux is ultimately exploited by measles virus to limit the death of infected cells and to improve viral particle formation. Whereas CD150 dependent virulent strains of measles virus are unable to induce the early CD46/GOPC dependent autophagy wave, they induce and exploit the late and sustained autophagy. Overall, our work describes distinct molecular pathways for an induction of self-beneficial sustained autophagy by measles virus.

## Author Summary

Autophagy is an evolutionarily conserved lysosomal dependent degradative pathway for recycling of long-lived proteins and damaged organelles. Autophagy is also an essential cellular response to fight infection by destroying infectious pathogens trapped within autophagosomes and plays a key role in the induction of both innate and adaptive immune responses. Numerous viruses have evolved strategies to counteract autophagy in order to escape from degradation or/and to inhibit immune signals. The kinetic and molecular pathways involved in the induction of autophagy upon infections might determine if cells would be able to control pathogens or would be invaded by them. We showed that measles virus (MeV) infection induces successive autophagy signallings in cells *via* distinct molecular pathways. A first autophagy wave is induced by the engagement of the MeV cellular receptor CD46 and the scaffold protein GOPC. A second wave is initiated after viral replication by the expression of the non-structural MeV protein C and is sustained overtime within infected cells thanks to the formation of syncytia. This sustained autophagy is exploited by MeV to limit the death of infected cells and to improve viral particle formation. We describe new molecular pathways by which MeV hijacks autophagy to promote its infectivity.

## Introduction

Measles is a highly infectious human disease caused by infection with measles virus (MeV), one of the most contagious human pathogens [1]. Measles infection takes place by the respiratory route and clinical symptoms include respiratory infection, fever, cough, coryza, conjunctivitis and the appearance of a generalized maculopapular rash, the hallmark of measles. Although MeV infection results in lifelong immunity, a transient but profound immunosuppression occurs by one to two weeks of infection and persists for several weeks [2]. MeV infection-induced complications essentially include secondary infections but also post-infectious encephalitis and subacute sclerosing panencephalitis (SSPE) [3], [4]. Despite the existence of an efficient vaccine based on attenuated MeV strains, recent measles outbreaks highlighted that this disease is still an important cause of mortality, especially among children in developing countries [1], [5]. The understanding of the biological interplay between attenuated versus virulent MeV strains and cellular components remains a challenge if we aim at developing tools to counter infection and use MeV as a vector for therapies.

MeV is a negative-stranded non-segmented RNA enveloped virus that belongs to the genus *Morbillivirus* of the *Paramyxoviridae* family [6]. Its genome encodes six structural proteins, the nucleoprotein MeV-N, a phosphoprotein MeV-P, an RNA polymerase MeV-L, the haemagglutinin MeV-H, the fusion protein MeV-F and the matrix protein MeV-M, and for two non-structural proteins not present within the virion, MeV-V and MeV-C. To date three different host-cell receptors for MeV-H attachment have been identified: CD46 which is expressed on all human nucleated somatic cells [7]; CD150 (also known as SLAM), expressed on immature thymocytes, activated B and T lymphocytes, macrophages and mature dendritic cells [8], and NECTIN-4 expressed on epithelial cells [9], [10]. Whereas MeV-H of clinical/virulent strains of MeV attach to CD150 or NECTIN-4, MeV-H of vaccine/attenuated-laboratory strains bind to either CD46, CD150 or NECTIN-4 [11]. Following attachment, conformational modification of the MeV-F protein leads to membrane fusion and virus entry at the host-cell plasma membrane, but also to syncytia formation, resulting from fusion of infected cells with uninfected cells [12]. Subsequently to fusion, the MeV ribonucleoprotein is delivered into the host-cell cytosol and viral mRNAs start to accumulate linearly as soon as 5 to 6 hours post infection before being exponentially increased until 24 hours [13]. MeV-V and MeV-C were shown to contribute to MeV replication by foiling innate antiviral immune responses [14], and newly generated ribonucleoproteins are assembled to bud from cell surfaces.

Among cell-host functions modulated upon MeV infection, we recently described macroautophagy, thereafter referred to as autophagy [15], [16]. Autophagy is a catabolic process essential for the maintenance of cellular homeostasis, through the elimination of otherwise deleterious cytosolic components, and for the recycling of metabolites [17], [18]. During autophagy, a cup-shaped isolated membrane, the phagophore, elongates within the cytosol up to generate an autophagosome vesicle that sequesters large portions of the cytoplasm. This content is ultimately degraded within autolysosomes, following maturation of autophagosome by fusion with a lysosome. At the molecular level, autophagy is a highly regulated process that involves numerous proteins including those encoded by autophagy-related (ATG) genes [19].

The autophagy machinery is used as a universal cell defence against intracellular microbes since it allows their delivery to degradative lysosomes [17]. Autophagy may also contribute to activate antiviral innate immunity [20], [21], as well as adaptive immune response by delivering virus-derived peptides for presentation by major histocompatibility complex (MHC) molecules to T lymphocytes [22], [23], [24]. Although upon a wide range of viral infections autophagosome formation is observed, few molecular details of viral interaction with the autophagy machinery are known [21]. Nevertheless, numerous viruses have evolved molecular strategies to counteract autophagy in order to escape this process, or even to exploit it to improve their own infectivity [25]. We have previously reported that the binding of an attenuated strain of MeV on CD46 induces the flux of autophagy. This induction relies on the molecular connection of one isoform of CD46, CD46-Cyt-1, to BECLIN-1 *via* the scaffold protein GOPC [16], [26]. Furthermore, we have shown that attenuated MeV can exploit autophagy through the physical interaction of the MeV-C protein with the autophagy-associated protein IRGM (immunity-related GTPase M) [27]. We wondered here how MeV regulates autophagy in the course of infection and how the virus exploits autophagy to its own advantage. We report that MeV infection induces successive autophagic signalling leading to a sustained increase of the autophagy flux. Whereas only attenuated MeV strains induce an early wave of autophagy dependent on CD46-Cyt1/GOPC, both attenuated and virulent strains induce and exploit a late but sustained productive autophagy wave.

## Results

### The attenuated strain of MeV induces two successive but independent waves of autophagy

**Figure 1:**
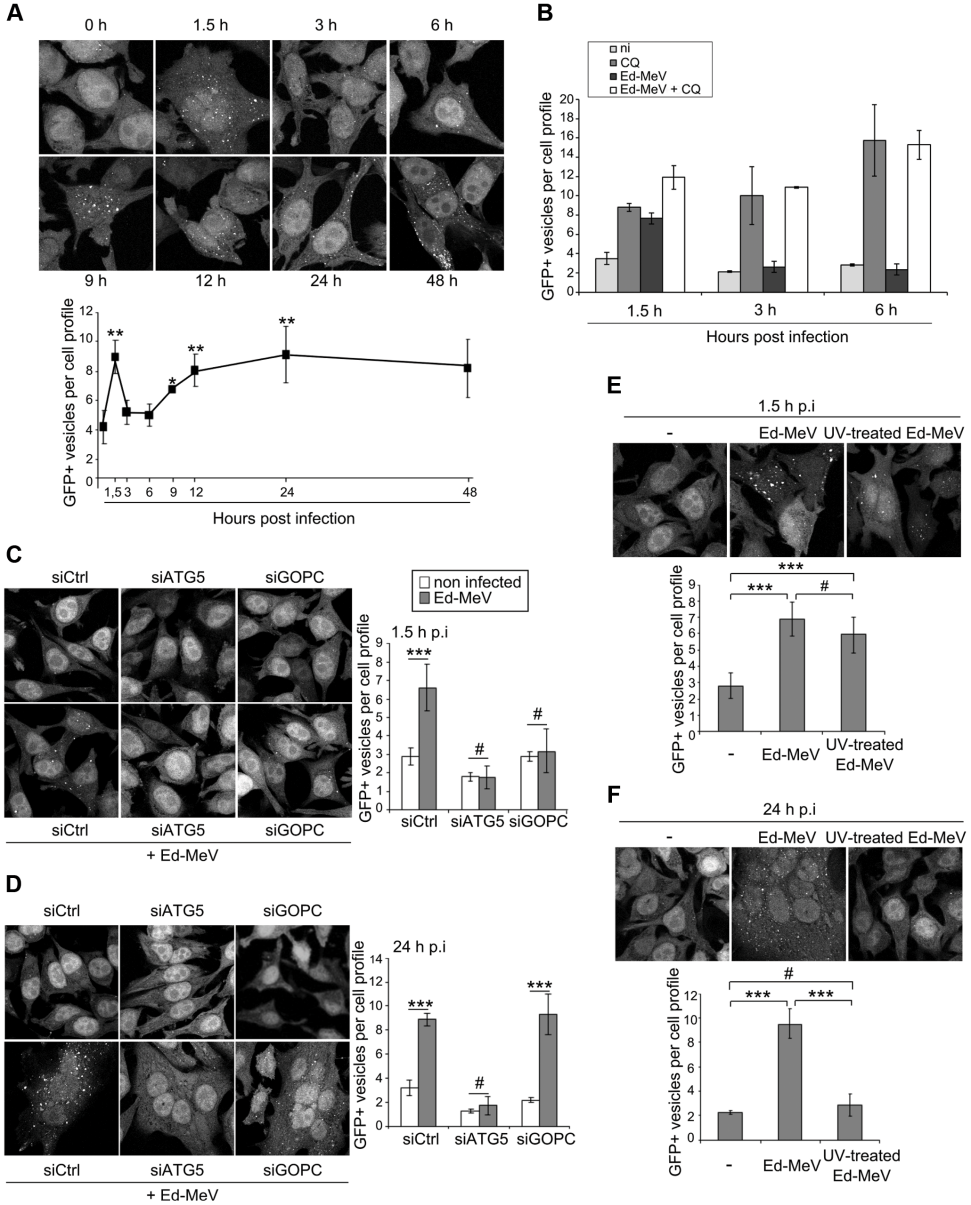
Attenuated strain of measles virus induces two waves of autophagy. (A) GFP-LC3-HeLa cells were infected with the attenuated strain of MeV Edmonston (Ed-MeV) at MOI 1. Autophagy was monitored by the numeration of GFP+ autophagosomes at the indicated period of time post infection. (B) GFP-LC3-HeLa cells were infected with Ed-MeV at MOI 1+/−75 µM Chloroquine (CQ) and autophagy was monitored at the indicated period of time post infection. (C–D) GFP-LC3 HeLa cells were treated with the indicated siRNA for 48 h and infected or not with Ed-MeV (MOI 1). Autophagy was monitored 1.5 h (C) or 24 h (D) post infection by the numeration of GFP+ autophagosomes. (E–F) GFP-LC3-HeLa cells were infected with Ed-MeV or UV-treated Ed-MeV (MOI 3) and 1.5 h (E) or 24 h (F) post infection (p.i.) the number of GFP+ vesicles per cell was assessed by confocal microscopy. For each experiment, representative profiles are shown and are accompanied by a graph representing the number of GFP+ vesicles per cell profile ( = GFP+ vesicles per one nucleus). For syncytia, the number of dots was normalized to the number of nuclei. Error bars, mean ± SD of three independent experiments for (A and C–F) and error bars, mean ± MD of two independent experiments for (B). Student's t test; ***p<0.005; **p<0.01; *p<0.05; #p>0.05.

We have previously reported that HeLa cell infection with the attenuated Edmonston (Ed)-MeV strain induces autophagy as soon as 1.5 hours post infection *via* the engagement of the CD46-Cyt-1/GOPC pathway [16]. We have also shown that autophagy was still detected by 24 hours of Ed-MeV infection [27]. Therefore, we examined autophagy kinetics upon Ed-MeV infection. GFP-LC3-HeLa cells were infected with Ed-MeV and autophagy was assessed at different time points by numerating GFP-LC3-labeled structures representing LC3-II-containing autophagosomes [28]. Strikingly, we found that Ed-MeV infection induces two successive waves of autophagosome accumulation (Fig. 1A). The first wave is induced as soon as 1.5 hours post infection, but this wave is transient and returned to basal level by 3 to 6 hours of infection. At these time points, autophagy was however still functional since the treatment of MeV-infected cells with chloroquine, which inhibits autophagosome recycling, still allowed the detection of an accumulation of autophagosomes (Fig. 1B and S1). This result suggests that following the transient early wave of autophagy, MeV does not actively inhibit autophagosome formation. Moreover, a second wave of autophagy is then induced, 9 hours after infection, and is sustained up to 48 hours post Ed-MeV infection (Fig. 1A). Similar waves of autophagy induction were observed by tracking LC3-II by western blot (Fig. S2). Of note, in the late time points, autophagosome accumulation was measured essentially in multinucleated cells, as MeV infection induces the massive formation of syncytia. Thus, for each experiment, the number of autophagosomes was normalized to the number of nuclei within a cell.

To investigate the molecular pathways underlining these two waves of autophagosome accumulation we used small interfering (si)RNAs to reduce the expression of putative key proteins. As expected, we found that the reduced expression of GOPC (Fig. S3) disrupted the early autophagosome accumulation, showing that early autophagy requires the expression of this scaffold protein (Fig. 1C, 1.5 h p.i.). In contrast, late autophagy was not affected by the reduced expression of GOPC, although as expected, the treatment of cells with si*ATG5* prevented autophagosome accumulation (Fig. 1D, 24 h p.i. and Fig. S3). Since the timing of MeV-induced late wave of autophagy correlated with the beginning of an efficient MeV replication (9 hours post infection) [13], we wondered whether virus replication was important for the induction of the late GOPC-independent autophagy wave. We found that infection of GFP-LC3-HeLa cells with a non-replicative ultraviolet-treated Ed-MeV (UV-Ed-MeV) still induced the early autophagosome accumulation (Fig. 1E). However, UV-Ed-MeV infection did not lead to the induction of the late wave of autophagy (Fig. 1F).

Altogether these results indicated that infection with an attenuated strain of MeV induces two successive waves of autophagy through distinct and uncoupled molecular pathways. An early one involves the cellular receptor CD46 and the scaffold protein GOPC, and a late one requires viral replication.

### The non-structural protein MeV-C is required to induce the late wave of autophagy during MeV infection

**Figure 2:**
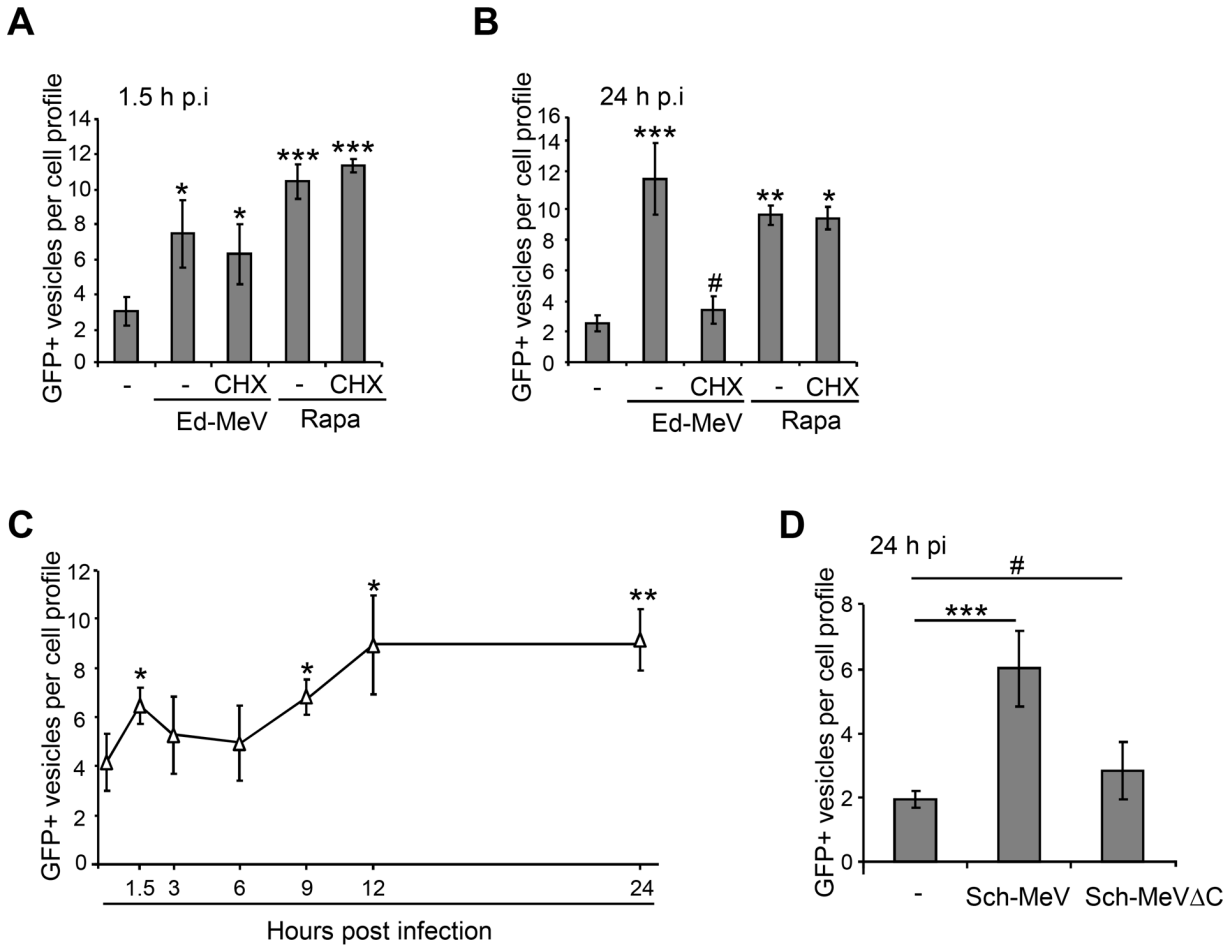
The MeV-C protein is involved in MeV-induced autophagy. (A–B) GFP-LC3-HeLa cells were infected or not with Ed-MeV (MOI 3) and treated or not with 0.5 µg/ml cycloheximide (CHX) during 1.5 h (A) or 24 h (B), in the presence of 125 nM rapamycin (Rapa) when indicated. (C) GFP-LC3-HeLa cells were infected with the attenuated strain of MeV Schwarz (Sch-MeV) at an MOI 1. Autophagy was monitored by the numeration of GFP+ autophagosomes at the indicated period of time post infection. (D) GFP-LC3-HeLa cells were infected with the attenuated strain of MeV Schwarz wild-type (Sch-MeV) or deficient for MeV-C expression (Sch-MeVΔC). 24 h post infection, the total number of GFP+ vesicles per cell was assessed by confocal microscopy in infected cells detected by staining for the viral nucleoprotein N (MeV-N). For each experiment, graph represents the number of GFP+ vesicles per cell ( = GFP+ vesicles per one nucleus). For syncytia, the number of dots was normalized to the number of nuclei. Error bars, mean ± SD of three independent experiments. Student's t test; ***p<0.005; **p<0.01; *p<0.05; #p>0.05.

The non-structural protein MeV-C is synthesised during the course of MeV infection. As we have recently shown that the single overexpression of MeV-C was sufficient to induce autophagy *via* an IRGM-dependent pathway [15], [27], we asked whether measles viral proteins synthesised during viral replication were indeed involved in late autophagy induction. To this end, we used 0.5 µg/ml cycloheximide which strongly prevented protein translation in the course of infection (Fig. S4), and autophagosome accumulation was monitored in GFP-LC3-HeLa cells infected with infectious Ed-MeV. Interestingly, whereas cycloheximide treatment did not modulate the early autophagy wave (Fig. 2A), it totally abolished the induction of the later one (Fig. 2B). The absence of autophagosome accumulation in 24 hours Ed-MeV-infected cells was not due to a limited availability of autophagy proteins due to cycloheximide treatment. Indeed, cells treated with rapamycin, an autophagy inducer, still induced autophagy in the presence of cycloheximide for the same period of time (Fig. 2A/B).

In order to determine whether MeV-C contributes indeed to autophagy induction during the course of infection, we then used a recombinant virus of the attenuated strain of MeV Schwarz (Sch-MeV) deficient for MeV-C expression (Sch-MeVΔC). First, we found that wild type Sch-MeV behaved as Ed-MeV since Sch-MeV infection induced two successive waves of autophagy, with similar kinetics than Ed-MeV infection (Fig. 2C). However, we found that in contrast to the wild type strain, Sch-MeVΔC did not induce autophagy 24 hours post infection (Fig. 2D). Thus, this result suggested that MeV-C expression is an essential prerequisite for an efficient induction of a second MeV-induced autophagy wave.

### Syncytia formation sustains autophagy in MeV-infected cells

**Figure 3:**
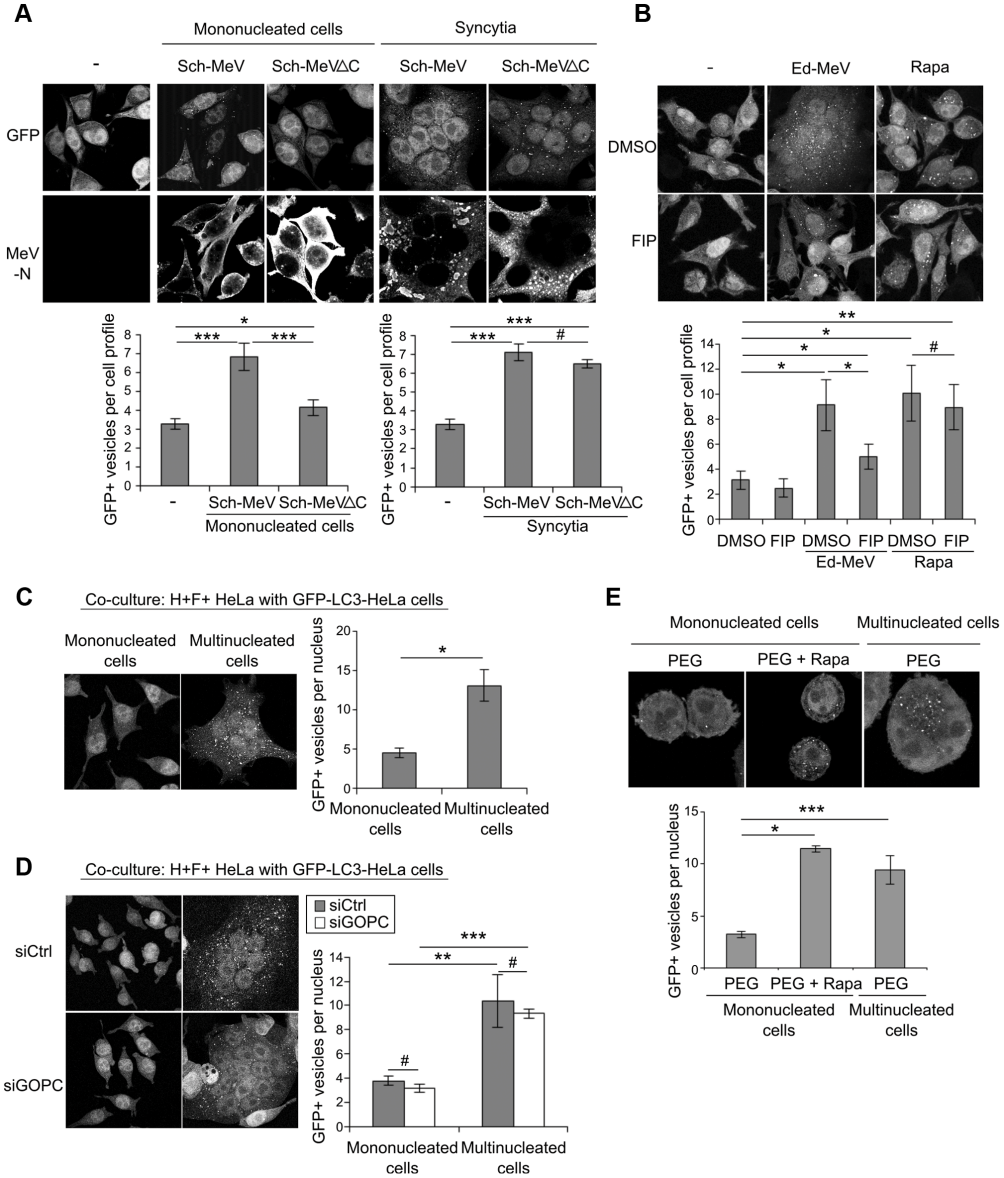
Syncytia formation induces autophagy in MeV-infected cells. (A) GFP-LC3-HeLa cells were infected with the attenuated strain of MeV Schwarz wild-type (Sch-MeV) or deficient for MeV-C expression (Sch-MeVΔC). 24 h post infection, the number of GFP+ vesicles within monocucleated or multinucleated cells was assessed by confocal microscopy in infected cells detected by staining for the viral nucleoprotein N (MeV-N). (B) GFP-LC3 HeLa cells were infected or not with Ed-MeV (MOI 3) or treated with rapamycin (Rapa) (125 nM), and treated or not with the FIP peptide (10 µg/mL). 24 h post infection, the number of GFP+ vesicles per cell was assessed by confocal microscopy. (C) HeLa cells were co-transfected with a vector encoding for the H protein of Ed-MeV and one encoding for the F protein. 24 h post transfection H/F-co-transfected HeLa cells (H^+^F^+^ HeLa) were co-cultured with GFP-LC3-HeLa cells to induce cell-cell fusion. The number of GFP+ vesicles within monocucleated or multinucleated cells was assessed by confocal microscopy after 18 h of co-culture. (D) GFP-LC3 HeLa cells and HeLa cells were treated with the indicated siRNA for 24 h. HeLa were then co-transfected with MeV-H/F as in (C) and co-cultured with siRNA treated GFP-LC3 HeLa cells. The number of GFP+ vesicles within monocucleated or multinucleated cells was assessed by confocal microscopy. (E) GFP-LC3 HeLa cells were treated with Polyethylene Glycol (PEG) and treated or not with 250 nM rapamycin (Rapa) for 2 h. The number of GFP+ vesicles within monocucleated or multinucleated cells was assessed by confocal microscopy 6 h after PEG treatment. For each experiment, representative profiles are shown and are accompanied by a graph representing the number of GFP+ vesicles per cell ( = GFP+ vesicles per one nucleus). For syncytia, the number of dots was normalized to the number of nuclei. Error bars, mean ± SD of three independent experiments. Student's t test; ***p<0.005; **p<0.01; *p<0.05; #p>0.05.

MeV infection massively induces syncytia formation. However, very few syncytia were detected upon Sch-MeVΔC infection (data not shown). We therefore wondered whether, beyond MeV-C expression, the formation of syncytia might contribute to the induction of autophagy upon MeV infection. We analysed autophagosome accumulation in either mononucleated or multinucleated cells 24 hours after infection with either Sch-MeV or Sch-MeVΔC. Autophagy was analysed in MeV-infected cells by numerating GFP-LC3+ vesicles exclusively in infected cells detected by MeV-N staining. Interestingly, whereas autophagy was detected in both mononucleated or multinucleated cells infected with Sch-MeV, autophagy was only detected in the rare multinucleated cells formed upon Sch-MeVΔC infection (Fig. 3A). Altogether, these results indicate that MeV-C is required to induce the late autophagy wave in infected cells that have not yet fused with other cells, but is dispensable in syncytia.

To further evaluate a role of syncytia formation in autophagy induction, wild type Ed-MeV-infected GFP-LC3-HeLa cells were cultured in the presence of a fusion inhibitory peptide (FIP) which inhibits syncytia formation without preventing individual infectious MeV particles entry within host cells [14]. Whereas the FIP treatment completely abolished MeV-induced syncytia formation (not shown), autophagy was still detected in 24 hours-infected mononucleated cells, although with a 50% reduction when compared to untreated infected cells (Fig. 3B). This reduction was neither due to a general effect of FIP on the autophagy process, since accumulation of autophagosomes by rapamycin was not affected by FIP treatment (Fig. 3B), nor to a decrease of viral protein synthesis as monitored by detection of similar levels of expression of MeV-N and MeV-P in the presence or not of FIP (Fig. S5).

To determine whether MeV replication within infected-syncytia was required to induce autophagy, we analysed autophagy in multinucleated cells in absence of MeV replication. First, MeV-H/F-co-transfected HeLa cells (Fig. S6A [29]) were co-cultured with GFP-LC3-HeLa cells leading to the cell-cell fusion *via* a viral H/F proteins dependent fusion process. In GFP^+^ multinucleated cells (those resulting from H^+^/F^+^ GFP-LC3^−^ cells which have fused with H^−^/F^−^ GFP-LC3^+^ cells), we found an increased number of autophagosomes, when compared with GFP^+^ mononucleated cells (Fig. 3C). The reduced expression of GOPC did not modulate autophagosome accumulation in these multinucleated cells (Fig. 3D). Moreover, multinucleated cells resulting from cell-cell fusion forced by polyethylene glycol (PEG) displayed an increased number of GFP-LC3^+^ autophagosomes (Fig. 3E). Thus, all together these results strongly suggested that in the course of MeV infection, the syncytia formation mediated by Ed-MeV-H/F viral proteins is sufficient to induce an autophagic signalling in multinucleated cells, independently of both viral replication and of the CD46/GOPC-dependent pathway.

### MeV proteins escape from degradation by sustained autophagy flux during MeV infection

**Figure 4:**
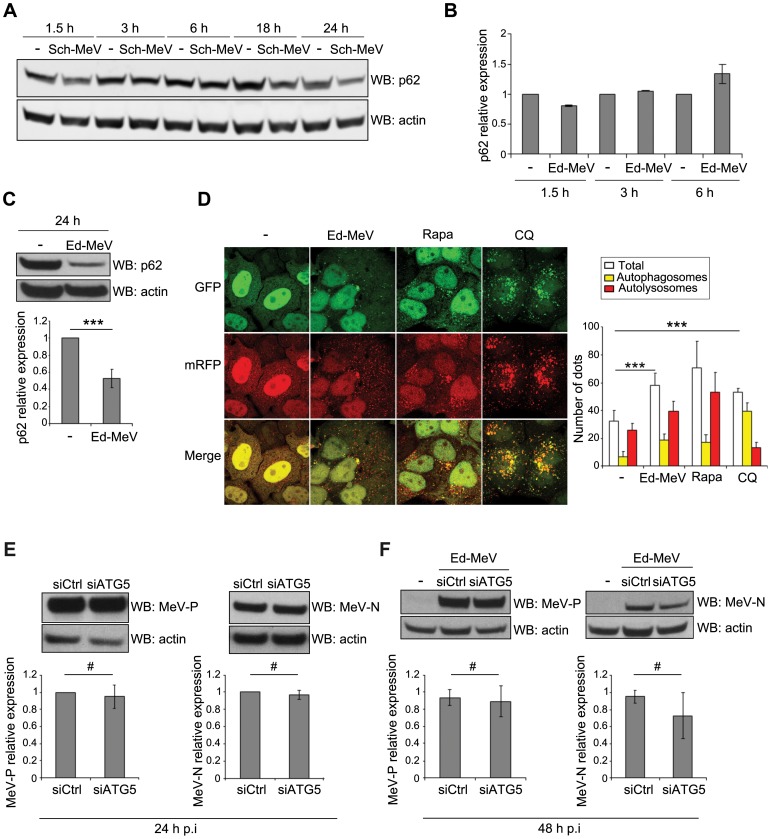
The late wave of autophagy induced by attenuated MeV is productive. (A) HeLa cells were infected with Sch-MeV (MOI 1). At the indicated time points post infection, cellular p62 expression was determined by western blot. (B) HeLa cells were infected with Ed-MeV (MOI 1). At the indicated time points post infection, cellular p62 expression was determined by western blot. The graph represents the intensity of p62 over actin expression normalized to the control condition (non infected). Error bars, mean ± MD of two independent experiments. (C) HeLa cells were infected with Ed-MeV (MOI 3). 24 h p.i., cellular p62 expression was determined by western blot. Representative results are shown and are accompanied by a graph representing the intensity of p62 over actin expression normalized to the control condition (non infected). (D) mRFP-GFP-LC3 HeLa cells were infected with Ed-MeV (MOI 3) or treated with 250 nM rapamycin (Rapa) or 75 µm Chloroquine (CQ) during 2 h. 24 h p.i., the total number of autophagic vesicles (mRPP+) and the number of autophagosomes (mRFP+/GFP+) were assessed by confocal microscopy. The number of autolysosomes was determined by subtracting the number of autophagosomes (mRFP+/GFP+) from the total number of vesicles (mRPP+). Representative profiles are shown and are accompanied by a graph representing the number of autophagic vesicles (white), autophagosomes (yellow) and autolysosomes (red) per nuclear profile for each condition. (E–F) HeLa cells were treated with the indicated si-RNA for 48 h and infected with Ed-MeV (MOI 3 (E) or MOI 0.1 (F)). 24 h (E) or 48 h (F) post-infection, cells were lysed and anti-N and anti-P western blot were performed to reveal MeV-N and MeV-P, respectively. Representative results are shown and are accompanied by a graph representing the intensity of MeV-N or MeV-P expression over cellular actin normalized to the control condition. (C–F) : error bars, mean ± SD of three independent experiments. Student's t test; ***p<0.005; **p<0.01; *p<0.05; #p>0.05.

Autophagy is a dynamic, multi-step process that can be modulated at several levels. An accumulation of autophagosomes can either reflect an increase of the autophagy flux, through the formation of *de novo* autophagosomes, or a reduced turnover of autophagosomes recycling, due to an inhibition of their fusion with lysosomes. We previously reported that the early CD46-Cyt-1/GOPC dependent induction of autophagy upon MeV infection resulted from an increase of the autophagy flux [16]. We confirmed this observation here by showing that the level of expression of the long-lived protein p62, a target of autophagy degradation, was reduced during the early wave of autophagy induced 1.5 hours post Ed-MeV or Sch-MeV infection (Fig. 4A/B). However, p62 expression was equivalent in 3–6 hours infected cells and in uninfected cells (Fig. 4A/B), the time points during which autophagy was not detected in MeV-infected cells (Fig. 1A).

We then asked whether the late and sustained accumulation of autophagosomes also resulted from an increased autophagy flux. We found a strong reduction of p62 expression in 24 hours Ed-MeV-infected cells suggesting an increase of the autophagy flux at this late time point (Fig. 4C). To further determine the reason of the late autophagosome accumulation upon infection, we used stably expressing mRFP-GFP-LC3-HeLa cells that allow for the distinction between autophagosomes (GFP^+^RFP^+^ puncta) and autolysosomes (GFP^−^RFP^+^ puncta) due to the quenched signal of the GFP in acidic compartments [30], [31]. Although we found an increased number of total autophagy vesicles in Ed-MeV-infected cells compared to control cells, an equivalent ratio of number of autophagosomes and autolysosomes was measured in infected cells and in control or rapamycin-treated cells (Fig. 4D). As expected, much more autophagosomes than autolysosomes were numerated in chloroquine-treated control cells, an inhibitor of autolysosome acidification (Fig. 4D). Thus, both the early and the late induction of autophagy following attenuated MeV infection induce *de novo* autophagosome formation and leads to the achievement of the autophagy process, without inhibition of autophagosome maturation. We therefore wondered whether this sustained autophagic flux degrades MeV proteins during the course of infection. We determined the level of expression of two viral proteins, the nucleoprotein MeV-N and the phosphoprotein MeV-P, in control cells and in cells treated with si*ATG5* in order to prevent autophagy. We found that autophagy competent or deficient cells expressed similar levels of MeV protein after 24 hours or 48 hours of infection (Fig. 4E/F). This result suggested that MeV proteins escape from autophagic degradation induced by MeV replication.

### MeV exploits the autophagy flux to replicate

**Figure 5:**
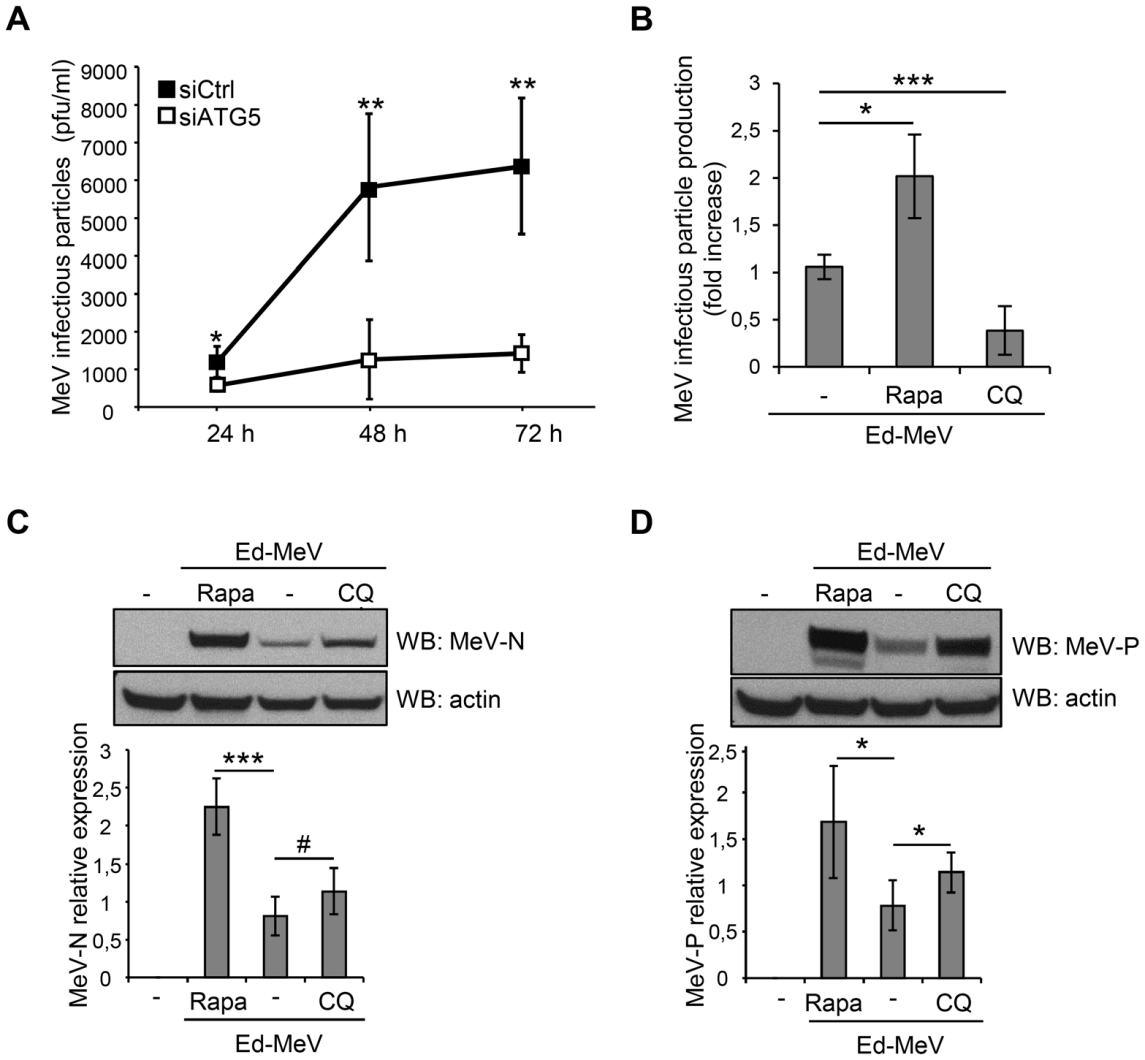
Ed-MeV exploits autophagy to replicate. (A) HeLa cells were treated with the indicated si-RNA for 48 h and infected with Ed-MeV (MOI 3). One, two or three days post infection infectious viral particles were titrated by plaque assay. (B–D) HeLa cells were treated or not with 125 nM rapamycin (Rapa) or 50 µM chloroquine (CQ) and infected with Ed-MeV (MOI 1 (B) or MOI 0.1 (C–D)). Three days post infection infectious viral particles were titrated by plaque assay (B) or 48 h post-infection, cells were lysed and MeV-N (C), MeV-P (D) expression were detected by western blot. (C–D) Representative results are shown and are accompanied by a graph representing the intensity of MeV-N (C) or MeV-P (D) expression over actin, normalized to the non-infected condition. For each experiment, error bars, mean ± SD of three independent experiments. Student's t test; ***p<0.005; **p<0.01; *p<0.05; #p>0.05.

Whereas several viruses take advantage of autophagy to replicate while inhibiting autophagosome maturation, MeV infection induces an increase of the autophagy flux. We wondered whether the autophagy flux was indeed required to improve viral particle production. First, we performed kinetics studies and, in accordance with our previous report [15], we found a strong impairment in Ed-MeV particle formation 24 hours to 72 hours post-infection in cells with a reduced expression of ATG5, as compared to control cells (Fig. 5A). Autophagy involvement in MeV particle formation was confirmed with the reduced expression of another autophagy essential gene *ATG7* (Fig. S7A), which also compromised Ed-MeV production (Fig. S7B).

We then looked at the incidence of experimental autophagy induction on MeV replication. We found that HeLa cells treated with the autophagy inducer rapamycin produced twice more infectious Ed-MeV particles than untreated cells (Fig. 5B). This result was corroborated when we analysed the expression of MeV proteins. We found that rapamycin treatment led to an increased level of expression of the two MeV proteins MeV-N and MeV-P (Fig. 5C/D).

Importantly, the inhibition of autophagosome maturation with chloroquine decreased the viral particle production, when compared to control cells, highlighting the importance of the autophagic flux for an efficient MeV replication in HeLa cells (Fig. 5B). However, chloroquine treatment did not have impact on the level of expression of MeV protein (Fig. 5C/D), confirming that these proteins are not targeted towards autophagic degradation. Altogether, these results indicated that MeV infection leads to a productive autophagy which is required for an efficient production of MeV infectious particles.

### Autophagy induced by MeV protects cells from death

**Figure 6:**
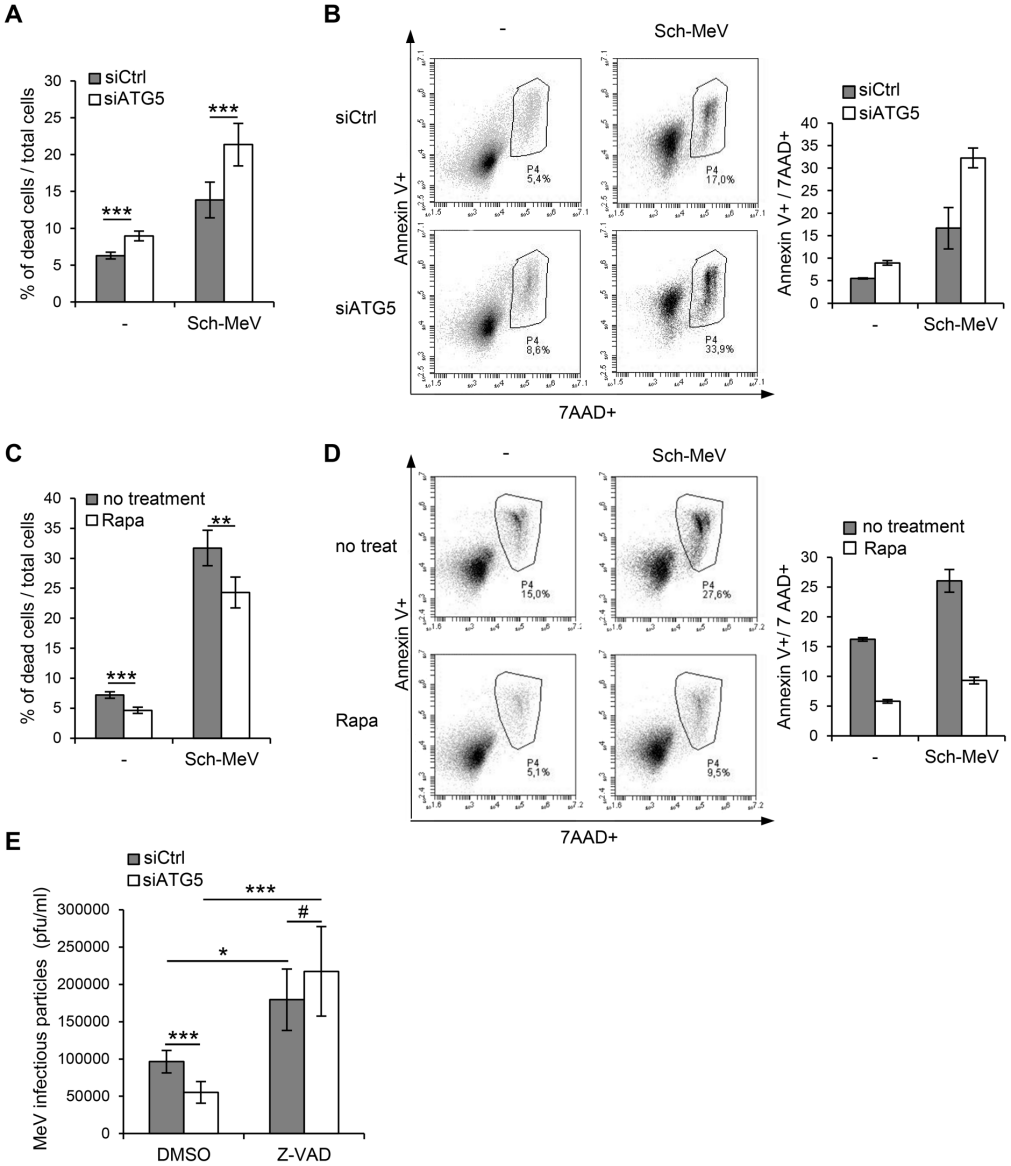
MeV-induced autophagy prevents cell death. (A–B) HeLa cells were treated with the indicated siRNA for 48 h and infected or not with Sch-MeV (MOI 0.1 for A and MOI 1 for B). (C–D) HeLa cells were treated or not with 250 nM rapamycin (Rapa) and infected or not with Sch-MeV (MOI 1). (A, C) 48 h post infection, cell death was analysed by trypan blue exclusion test. Graphs represent the percentage of dead cells compared to the number of total cells. Error bars, mean ± SD of two independent experiments made in triplicate (A) and two independent experiments made in duplicate (C).(B, D) 48 h post infection, cell death was analysed by Annexin V and 7AAD staining. Representative results of two experiments made in duplicate are shown. (E) HeLa cells were treated with the indicated siRNA for 48 h and infected with Sch-MeV (MOI 0.1) and treated with DMSO or with 50 µM Z-VAD. 48 h p.i, infectious viral particles were titrated by plaque assay. Error bars, mean ± SD of three independent experiments. Student's t test; ***p<0.005; **p<0.01; *p<0.05; #p>0.05.

As autophagy has no direct impact on viral protein synthesis, we wondered whether MeV could take benefit from autophagy by extending infected cell survival. We found that autophagy inhibition using *siATG5* increased death of cells infected by Sch-MeV as measured by cell numeration (Fig. 6A). We confirmed this result by looking at the percentage of late apoptotic cells/dead cells determined by the double labeling for Annexin V and 7-Amino-Actinomycin (7AAD) and found twice more Annexin V+/7AAD+ in si*ATG5*-treated cells than in control cells upon Sch-MeV infection (Fig. 6B). In contrast, the experimental induction of autophagy with rapamycin prior infection was found to protect Sch-MeV-infected cells from cell death (Fig. 6C/D). Moreover, the reduced expression of ATG5 increased Sch-MeVΔC-infected cell death (Fig. S8), but more slightly than wild type Sch-MeV (Fig. 6A). However, autophagy promotion with rapamycin protected efficiently Sch-MeVΔC-infected cells from death (Fig. S8). Taken together, these results strongly suggested that autophagy induced by MeV could contribute to protect infected cells from death what contributes to improve infectivity. Corroborating this hypothesis, we found that the inhibition of apoptosis, using the pan-caspase inhibitor Z-VAD, improved MeV particle production by 48 hours-infected cells (Fig. 6E). Furthermore, the prevention of autophagy in Z-VAD-treated cells did not modulate MeV production (Fig. 6E), suggesting that the main function of autophagy in the course of MeV infection is to delay MeV-induced apoptosis.

### A virulent strain of MeV induces and exploits the autophagic flux

**Figure 7:**
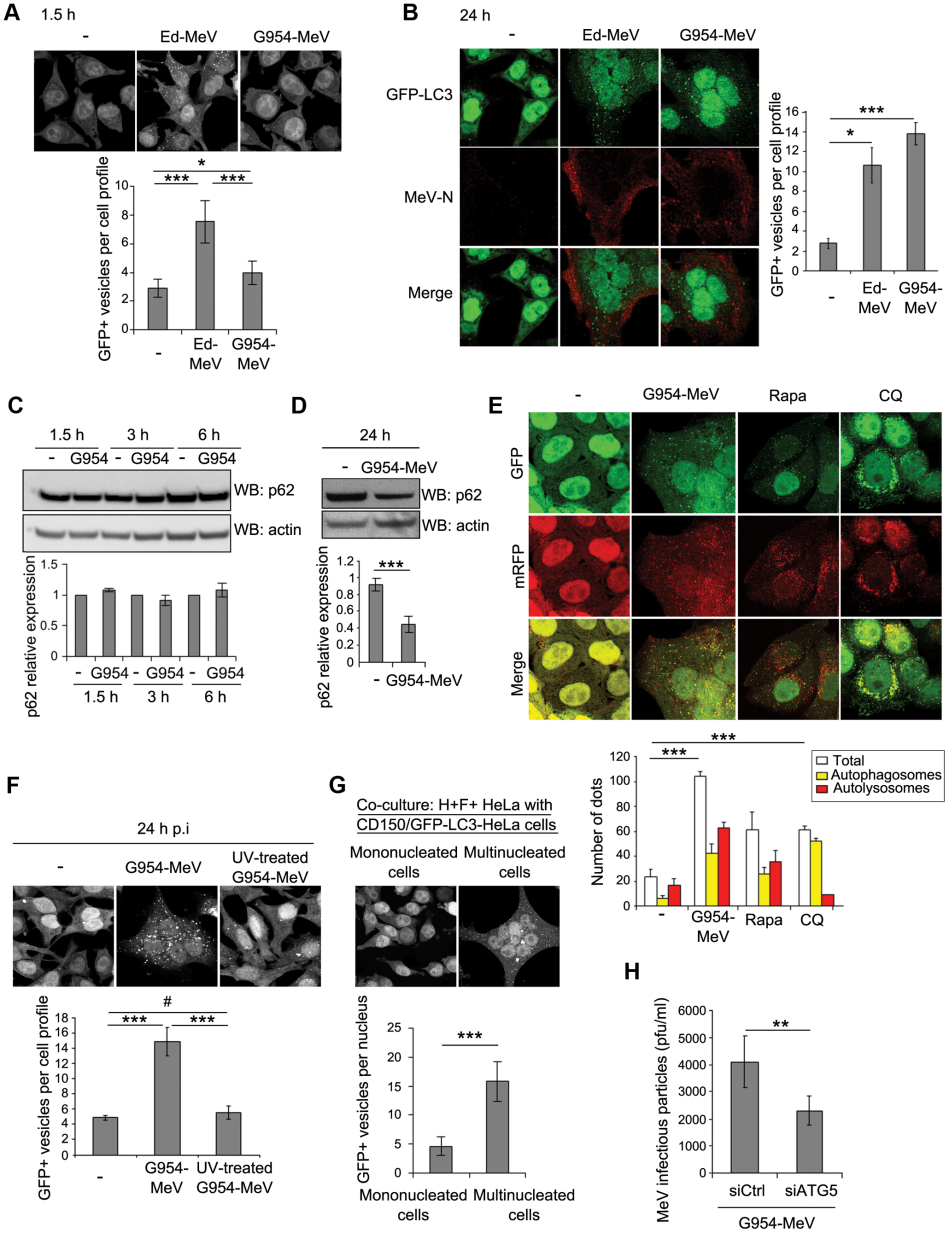
A virulent strain of MeV induces and exploits autophagy. (A) CD150-stably-expressing GFP-LC3-HeLa were infected with Ed-MeV at MOI 1 or with the virulent strain of MeV G954 (G954-MeV) at MOI 1. Autophagy was monitored by the numeration of GFP+ autophagosomes 1.5 h p.i.. (B) CD150-transfected GFP-LC3-HeLa cells were infected with Ed-MeV at MOI 1 or with the virulent strain of G954-MeV at MOI 0.1. Autophagy was monitored by the numeration of GFP+ autophagosomes 24 h p.i in infected cells detected by staining for the viral nucleoprotein N (MeV-N). (C) CD150-stably-expressing HeLa cells were infected with G954-MeV (MOI 1). At the indicated time points, cellular p62 expression was determined by western blot. Representative results are shown and are accompanied by a graph representing the intensity of p62 over actin expression normalized to the control condition (non infected). Error bars, mean ± SD of two independent experiments. (D) CD150-transfected HeLa cells were infected with G954-MeV (MOI 0.1). 24 h p.i., cellular p62 expression was determined by western blot. Representative results are shown and are accompanied by a graph representing the intensity of p62 over actin expression normalized to the control condition (non infected). (E) CD150-transfected mRFP-GFP-LC3 HeLa cells were infected with G954-MeV (MOI 0.1) or treated with 250 nM Rapa or 75 µm CQ during 2 h. 24 h p.i., the autophagic vesicles were analysed as in figure 4D. (F) CD150-transfected GFP-LC3-HeLa cells were infected with G954-MeV or UV-treated G954-MeV (MOI 0.1). 24 h post infection, the number of GFP+ vesicles per cell was assessed by confocal microscopy. (G) HeLa cells were co-transfected with a vector encoding for the H protein of KA-MeV (a CD150-dependent strain) and one encoding for the F protein of Ed-MeV. Autophagy was determined as in figure 3C. (H) CD150-transfected HeLa cells were treated with the indicated si-RNA for 48 h and infected with G954-MeV (MOI 0.1). Two days post infection, infectious viral particles were titrated by plaque assay. For each experiment (except for (C)), error bars, mean ± SD of three independent experiments. Student's t test; ***p<0.005; **p<0.01; *p<0.05; #p>0.05.

We then investigated whether a virulent strain of MeV, which does not bind CD46 but CD150 to infect cells [8], might also induce the two waves of autophagy during infection, and exploit autophagy in HeLa cells. As expected, we did not detect autophagy in HeLa cells incubated with the virulent G954-MeV strain, since these cells do not express CD150 (Fig. S9) [8]. However, interestingly we found only a marginal accumulation of GFP+ dots in CD150-GFP-LC3-HeLa cells, 1,5 hours after infection (Fig. 7A and Fig. S10). By contrast, a strong increase of the number of autophagosomes was observed 24 hours after G954-MeV infection (Fig. 7B). These dots corresponded to autophagosomes since their accumulation was abolished in cells with a reduced expression of the essential gene for autophagy *ATG5* (Fig. S11). Additionally, we did not observe a decrease of p62 expression 1,5 hours post G954-MeV infection, whereas its expression drops off by 24 hours of infection (Fig. 7C/D). Moreover, tracking RFP-GFP-LC3 in infected CD150-HeLa cells confirmed that the autophagy flux is increased in 24 hours G954-MeV-infected cells (Fig. 7E). Thus, these results suggested that virulent CD150-dependent CD46-independent MeV strain infection does mostly induce the productive late wave of autophagy.

Additionally, we found that UV-G954-MeV and cycloheximide treatment prevented autophagosome accumulation in 24 hours G954-MeV-infected CD150-HeLa cells, indicating that, as for attenuated strains, neo-synthesized viral proteins are required for autophagy infection by a virulent MeV strain (Fig. 7F and S12). Moreover, virulent H/F co-expression on HeLa cells (Fig. S6B) co-cultured with CD150-GFP-LC3 HeLa cells was sufficient to induce an autophagic signalling in multinucleated cells, independently of viral replication (Fig. 7G). Finally, we found that virulent MeV replicated less efficiently in si*ATG5*-treated cells than in control cells (Fig. 7H). Altogether, these results indicated that a virulent MeV strain unable to induce the first CD46-dependent autophagic wave, induces and exploits the late autophagic wave to replicate.

## Discussion

We found that infection with attenuated MeV induces two successive waves of autophagy *via* distinct molecular pathways. We have previously described the attenuated MeV receptor CD46-Cyt-1 as a pathogen receptor able to induce an early autophagy flux, subsequently to pathogen detection, *via* its association with the scaffold protein GOPC, which relies to the autophagosome formation complex BECLIN 1/VPS34 [16]. We show here that this autophagy induction is very transient as it probably stops after CD46/GOPC-mediated autophagic signalling following virus entry. Interestingly, virulent strains do not bind to CD46 and therefore are unable to induce the CD46-dependent early autophagy wave. Instead, virulent strains bind to CD150. CD150 was reported to recruit the autophagy-associated molecules BECLIN 1 and VPS34 to the phagosome of macrophages which uptake gram-negative bacteria [32]. However, whether CD150 engagement regulates the autophagy process has not been observed. Our results indicate that CD150 does not induce autophagy (or very marginally) upon virulent MeV entry. Thus, an attractive hypothesis would be that the first autophagy wave induced by attenuated/vaccinal MeV strains, but not triggered by virulent/clinical ones, could contribute to the attenuation of MeV infectivity, an issue that remains to be investigated. An immune function resulting from CD46-induced autophagy could be to facilitate intravesicular TLR engagement. Indeed in plasmacytoid dendritic cells, TLR7 gains access to viral-replication intermediates through autophagy to induce antiviral type I interferon (IFN-I) production [20]. Interestingly, in B cells, TLR9 is recruited to autophagosomes upon B cell receptor (BCR) internalization to enhance B cell activation [33]. Furthermore, it was shown that CD46-binding adenoviruses are routed in a pathway that allows TLR9-dependent IFN-I induction, whereas adenoviruses using CAR as cellular receptor does not [34]. Thus, the immediate autophagy induction mediated by CD46 might confer an early response towards CD46-binding MeV strains, protecting cells from massive replication by producing IFN-I prior to extended MeV replication, and contributing to their attenuation. HeLa cells being poor producers of IFN-I, in our experimental setup attenuated MeV can replicate efficiently. It will be of interest to determine whether autophagy induced by CD46-binding viruses is involved in early IFN-I induction.

After few hours of infection, a new autophagy flux is induced, which is sustained over time. This process is independent of a CD46-Cyt-1/GOPC signalling, but requires viral replication and relies on the expression of the C protein of MeV. Importantly, in HeLa cells, for attenuated and virulent strains of MeV, we demonstrated that this second wave of autophagy is manipulated by MeV to increase infectious viral particle formation. We described recently the autophagy-associated protein IRGM as being a cellular target of MeV for both the induction of autophagy 24 hours post-infection (what correspond to the second autophagy wave), and to improve MeV infectivity [15]. Moreover, we have reported that the single expression of MeV-C, which can interact with IRGM, was sufficient to induce autophagy, through an IRGM dependent pathway [15]. Here, we extended these observations by showing that MeV deficient for the expression of the C protein (MeVΔC) does not induce autophagy in infected mononucleated cells. These results confirmed a prominent role of the MeV-C protein in the induction of the second wave of autophagy, subsequently to MeV replication. The exact role of MeV-C in MeV-induced autophagy requires further investigations. We have previously shown that the MeV-C partner IRGM can interact with several other human autophagy-associated proteins, ATG5, ATG10, LC3C and BIF-1, which could be involved in MeV-induced autophagy, consequently to C expression [15], [35]. Interestingly, MeV-C was also shown to interfere with host defence mechanisms by dampening antiviral IFN-I activation through the downregulation of viral RNA synthesis in order to limit their detection by IFN-I-inducing cytosolic receptor such as RIG-I or MDA5 [36], [37]. We highlight here an unrevealed role of the C protein in autophagy induction upon MeV infection, which is ultimately used by the virus to improve its infectivity.

Strikingly, whereas the expression of MeV-C is required for the induction of the late autophagy wave in infected mononucleated cells, its expression is not critical for the induction of autophagy in syncytia. Indeed, we found that whereas Sch-MeVΔC does not induce autophagy in mononucleated cells, autophagy was still observed in syncytia. Interestingly, it has recently been suggested that viruses of the *Morbillivirus* genus including MeV, could induce autophagy through a fusogenic dependent mechanism which requires the coexpression of MeV-F and MeV-H proteins [38]. Using an alternative approach to analyse autophagy, we found an increase of autophagosomes in multinucleated cells formed through a MeV-H/F proteins-mediated process. Furthermore, we found that the forced fusion between cells promoted by PEG-treatment induces autophagy. Thus, the plasma membrane perturbations/damages resulting from cell-cell fusion could be sufficient to trigger an autophagy signalling, with functions to eliminate/recycle excessive/redundant/damaged cytosolic materials within newly formed multinucleated cells. The upstream signals inducing autophagy subsequently to the fusion between cells remain to be investigated. Interestingly, it was proposed that autophagy could play a role in syncytia formation [38]. Thus, an attractive hypothesis would be that during MeV infection, the MeV-C protein induces autophagy in infected cells through an IRGM-dependent pathway, which could contribute to the facilitation of the syncytia formation. In turn, through a fusogenic process, syncytia formation could further increase autophagy in multinucleated cells leading to a positive feedback loop of autophagy induction, maintained by MeV-C expression. In accordance with this hypothesis, the number of syncytia is strongly reduced upon infection with Sch-MeVΔC compared with wild type Sch-MeV (data not shown). Thus, whereas the first wave of autophagy triggered by the CD46-Cyt-1/GOPC pathway would concern exclusively the primary infected cells by infectious attenuated viral particles, the second wave is induced following MeV-C expression and would be maintained over time by syncytia formation.

MeV-induced syncytia were reported to be dynamic entities with an unusual extended life span [39]. Interestingly, we found that autophagy contributes to protect MeV-infected cells from apoptotic cell death. Thus autophagy induction in syncytia could delay MeV-induced cell death, and contribute to an efficient spreading of the virus. Indeed, we report that inhibition of apoptosis strongly facilitates MeV replication, what is not anymore modulated by additional autophagy inhibition. Furthermore, in support of this hypothesis MeVΔC, which does not induce autophagy over basal level in mononucleated cells, presents a growth defect and induces more apoptosis in infected cells than wild type virus [40]. Thus, one way by which MeV exploits autophagy is by protecting infected cells from cell death which otherwise could limit viral replication and propagation of numerous infectious viral particles. Other viruses were recently described to manipulate autophagy in order to prevent cell death, as the human flavivirus dengue virus type 2 and the Chikungunya virus [41], [42].

MeV infection induces an increase of the autophagy flux. However, MeV proteins are not degraded by autophagy what suggests that MeV proteins would escape from targeting towards autophagosomes. Indeed, colocalization between GFP-LC3 and the MeV-N protein was not observed. Furthermore, it has been recently reported that the *Morbillivirus* replication complex and autophagosomes do not colocalize [38]. However, we have shown that maturation of autophagosomes is necessary to promote the formation of infectious viral particles. Beyond delaying death of infected cells, productive autophagy could contribute to the generation of an excess of metabolites used by MeV to optimize its replication. Autophagy induced by Dengue virus infection is used to regulate cellular lipid metabolism to generate ATP and to promote viral replication [43]. As we found that MeV proteins synthesis is not modulated by autophagy, nucleic acids and/or lipids generated from autophagy-mediated recycling could be primary metabolites exploited to improve MeV replication and/or assembly. Alternatively, the different autophagy signalling triggered in the course of MeV infection might be required to coordinate different steps of the virus cycle. Indeed, productive autophagy has been shown to facilitate poliovirus replication, which gradually gets benefit of the complete autophagic process : whereas autophagosome formation is involved in the viral RNA synthesis and early phases of the virus cycle, the acidification of the autolysosomes contributes to the final maturation of virus particles [44]. Similarly, autophagy impacts on the final maturation of infectious Dengue virus particles formation, as inhibition of autophagy leads to the production of noninfectious particles [45]. The contribution of the different autophagy signallings in MeV maturation remains to be investigated.

Our work describes the induction of successive molecular pathways contributing to autophagy in response to an infection by attenuated MeV. Whereas an early wave of autophagy induction is triggered by the host recognition of the pathogen, *via* CD46-Cyt-1/GOPC, a later one seems to be the result of a direct interaction of viral proteins with the autophagy machinery, and is sustained within syncytia. The sustained wave is exploited by attenuated as well as virulent strains of MeV to promote the production of infectious viral particles. The complete understanding of the complex relationship between autophagy and MeV might allow a better understanding of the contribution of autophagy for the attenuated or virulent characters of a pathogen, and a better usage of MeV-derived vaccine for therapy.

## Material and Methods

### Ethics statement

The experiments in this article were performed at Biological Safety Level 2 in accordance with the regulations set forth by the national French committee of genetics (commission de génie génétique).

### Cell culture

HeLa, GFP-LC3-HeLa and mRFP-GFP-LC3 HeLa cells were maintained in RPMI 1640, Vero cells were maintained in DMEM. All the media were supplemented with 50 µg/mL gentamicin and 10% fetal bovine serum (FBS).

### MeV strains and tiration by plaque assay

Ed-MeV was obtained from ATCC and G954-MeV was kindly provided by B. Horvat (INSERM U1111, France). HeLa cells were infected with MeV at the indicated MOI. After the indicated period of infection, cells were submitted to 5 cycles of freezing at −80°C and defrosting at 37°C and infectious viral particles were quantified by limiting dilution on confluent Vero cells.

### SiRNA transfection

Smartpool siRNA targeting ATG5, ATG7 and GOPC, as well as control siRNA (AUACCUAACUGAUGAGACCUU) were from Dharmacon (Perbio, Brebières, France). 0.1×10^6^ HeLa, GFP-LC3 HeLa cells were plated in 6-well plates 24 hours prior to transfection with 100 pmol si-RNA using Lipofectamine RNAiMAX (Invitrogen) according to manufacturer's instructions. Protein expression level was assessed by western-blot three days post transfection.

### CD150 transfection

HeLa, GFP-LC3-HeLa and mRFP-GFP-LC3 HeLa cells were transfected with the plasmid pCAGGS-CD150, kindly provided by Y. Yanagi (Japan) [8], using lipofectamine 2000 (Invitrogen) according to manufacturer's instructions. 24 h post transfection, CD150 expression was checked by FACS analysis after a staining using an anti-CD150-PE antibody and as a negative control an isotype antibody.

### Generation of stable cells expressing CD150

The lentiviral *peGAET-ires-puro* expression plasmids was a gift of P. Mangeot (ENS-Lyon, INSERM U1111, Lyon, France). To generate peGAET-cd150-ires-puro plasmid, cd150 sequence was amplified by PCR and replaced the tTA sequence in peGAET-ires-puro into the EcoR1 and XhoI restriction sites. Viral particles were commonly produced by cotransfection of 293T cells with lentiviral peGAET-cd150-ires-puro plasmid and the helper plasmids encoding the proteins required for vector packaging (Plateau AniRA Vectorologie UMS3444, US8). Supernatant was collected at days 2 post-transfection, filtered and concentrated by ultracentrifugation. GFP-LC3-HeLa and HeLa were transduced with concentrated viral particles in the presence of 8 µg/ml polybrene (Sigma). 48 h post transduction, cells were treated with 1 µg/ml puromycin for 10 days. Surviving clones were expanded in 1 µg/ml puromycin and analysed for stable integration of the transgene and expression of CD150 protein by flow cytometry.

### MeV-H and MeV-F transfection

HeLa cells were co-transfected with the plasmid pCXN2-F (Edmonston strain), and either with the plasmid pCXN2-H (Edmonston strain) or pCXN2-H (KA strain) [37] using lipofectamine 2000 (Invitrogen) according to manufacturer's instructions. 24 h post transfection, MeV-H and MeV-F expression were checked by FACS analysis using specific primary antibodies.

### Polyethylene glycol (PEG) treatment

GFP-LC3-HeLa cells were seeded in 25 cm^2^ flask to be confluent the next day, in RPMI 10% FBS in absence of antibiotics. A PEG solution was prepared by mixing 10 g of autoclaved PEG 6000 (Sigma) with 10 ml of RPMI without FBS. The cells were washed twice with warmed PBS and then incubated 10 min at 37°C with PEG. The PEG was then removed and RPMI free of serum was added progressively onto the cells. This RPMI was then removed and replaced by 5 ml of RPMI 10% FBS for 4 hours. Finally, the cells were detached with Versene (Invitrogen) and plated in 24 well-plates on cover slips coated with Poly-L-Lysine and treated with rapamycin for 2 hours.

### Western blot

Cells were lysed with lysis buffer (PBS-1×, NP40 1% or 0.5% and protease inhibitor (Roche)). Soluble proteins were separated by SDS-PAGE (NuPAGE Novex Tris-Acetate Mini Gels, Invitrogen) and transferred to nitrocellulose membranes with iblot Gel Transfer System (Invitrogen). Primary antibodies, anti-Actin (mouse monoclonal), anti-MeV-N (mouse monoclonal), anti-MeV-P (mouse monoclonal or rabbit polyclonal), anti-p62 (mouse monoclonal), secondary HRP linked anti-rabbit or HRP linked anti-mouse antibodies were used and antigen-antibody complexes were visualized by enhanced chemiluminescence.

### Confocal microscopy for autophagy analysis

After the indicated treatments, GFP-LC3-HeLa or mRFP-GFP-LC3 HeLa cells were fixed with 4% paraformaldehyde. For Measles nucleoprotein staining, an anti-MeV-N antibody (mouse monoclonal, clone 120 produced in the lab) was used at 10 µg/mL, followed by secondary antibody conjugated to Alexa Fluor 568. Cells were analysed using a Confocal Axioplan2 LSM510 microscope (Zeiss, Göttingen, Germany) equipped with the LSM 510 META system (Zeiss) and mounted with an Axioscope 63× oil immersion lens (Zeiss). The number of GFP + was numerated from one single plan section per cell and normalized to the number of nuclei. In the legend, a cell profile means “per nucleus” because for syncytia, number of GFP+ vesicles was normalized to the number of nuclei. In each case, number of GFP+ vesicles was numerated from 100 to 200 cells for each experiment.

### Trypan blue exclusion test of cell viability

Cells were mixed with 0.4% trypan blue (Gibco) and unstained (viable) and stained (nonviable) cells were numerated on a Bürker microscope slide (Marienfeld). For each condition, at least 100 cells have been numerated.

### Annexin V and 7AAD staining

Cells were stained with the PE Annexin V Apoptosis Detection Kit I (BD Pharmingen) according to manufacturer's instructions and cells were analysed by using an Accuri C6 flow cytometer and the C Flow software.

### Antibodies

Anti-MeV-N (mouse monoclonal, clone 120), MeV-H (mouse monoclonal, clone 55), anti-MeV-F (mouse monoclonal, clone Y503), and anti-GOPC (GOPC-GST rabbit anti-serum) were produced in the lab, anti-MeV-P (mouse monoclonal 49.21 or rabbit polyclonal J37171) were kindly provided by D. Gerlier (INSERM U1111, France). Anti-Actin (A2066), anti-ATG5 (A0856), anti-MAP1LC3B (L7543) and anti-ATG7 (A2856) were from Sigma (St Louis, Mo, USA). Anti-rabbit HRP (NA 934) or anti-mouse HRP (NA 931) were from Amersham Biosciences (Uppsala, Sweden). Anti-p62 (SQSTM1 (D-3): sc-28359) was from Santa Cruz Biotechnology (California, USA). Anti-mouse Alexa Fluor 568 was purchased from Invitrogen (Molecular Probes). Anti-CD150-PE (559592) was from BD Pharmigen and isotype (IOTest IgG2a (Mouse)-FITC/IgG1 (Mouse)-PE) was from Beckman Coulter (Immunotech SAS, Marseille, France).

### Reagents

Pharmacological reagents used were cycloheximide (C4859, Sigma), rapamycin (Calbiochem), chloroquine (C6628, Sigma) and Z-Val-Ala-DL-Asp(OMe)-fluoromethylketone (Z-VAD) (N-1560, Bachem).

## Supporting Information

Figure S1 **Effect of chloroquine during Ed-MeV infection.** GFP-LC3 HeLa cells were infected with Ed-MeV at MOI 1 and/or treated with 75 µm Chloroquine (CQ). Autophagy was monitored by the numeration of GFP+ autophagosomes at the indicated period of time post infection. CQ was maintained all along during the culture. Representative profiles are shown and are accompanied by a graph representing the number of GFP+ vesicles per cell profile. Error bars, mean ± MD of two independent experiments.
(TIF)

Figure S2 **LC3-II expression during Ed-MeV infection.** GFP-LC3-HeLa cells were infected with Ed-MeV (MOI 1). At the indicated time points post infection, autophagy was assessed by measuring LC3 conversion by western blot.
(TIF)

Figure S3 **Efficiency of siRNA.** HeLa cells were treated with the indicated siRNA. 48 h later, the expression of the respective proteins were assessed by western blot.
(TIF)

Figure S4 **Efficiency of cycloheximide treatment.** GFP-LC3 HeLa cells were infected with attenuated Ed-MeV (MOI 3) in the presence of 1 µg/ml or 0.5 µg/ml cycloheximide (CHX). 24 h post-infection, cells were lysed and anti-N and anti-P western blot were performed to reveal MeV-N and MeV-P, respectively. Representative results are shown and are accompanied by a graph representing the intensity of MeV-N or MeV-P expression over cellular actin, and normalized to the control condition (DMSO treatment). Error bars, mean ± SD of two independent experiments. Student's t test, ***p<0.005; *p<0.05.
(TIF)

Figure S5 **FIP treatment does not affect MeV viral protein synthesis.** HeLa cells were infected or not with Ed-MeV (MOI 3) and treated or not with the FIP peptide (10 µg/mL). 24 h post-infection, cells were lysed and anti-N and anti-P western blot were performed to reveal MeV-N and MeV-P, respectively. Representative results are shown and are accompanied by a graph representing the intensity of MeV-N or MeV-P expression over cellular actin normalized to the control.
(TIF)

Figure S6 **Expression of MeV-H and MeV-F on co-transfected cells.** (A) HeLa cells were co-transfected with a vector encoding for the H protein of Ed-MeV (A) or the H protein of KA-MeV (B), and one encoding for the F protein (A and B). 24 h post transfection, expression of MeV-H and MeV-F was measured by FACS analysis.
(TIF)

Figure S7 **Ed-MeV particle formation is reduced in si** ***ATG7*** **-treated cells.** (A) the efficiency of si*ATG7* treatment is shown by western blot, for one representative experiment. (B) HeLa cells were treated with the indicated siRNA for 48 h and infected with Ed-MeV (MOI = 2). Two days post infection viral particles were titrated by plaque assays. Error bars, mean ± SD of six independent experiments. Student's t test; ***p<0.005.
(TIF)

Figure S8 **Autophagy protects Sch-MeVΔC infected cells from death.** (A) HeLa cells were treated with the indicated siRNA for 48 h and infected or not with Sch-MeVΔC (MOI 0.1). (B) HeLa cells were treated or not with 250 nM rapamycin (Rapa) and infected or not with Sch-MeVΔC (MOI 1). (A, B) 48 h post infection, cell death was analysed by trypan blue exclusion test. Graphs represent the percentage of dead cells compared to the number of total cells. Error bars, mean ± SD of two independent experiments made in triplicate (A) and two independent experiments made in duplicate (B).
(TIF)

Figure S9 **The virulent strain of measles virus does not induce autophagy in HeLa cells.** GFP-LC3 HeLa cells were infected with attenuated Ed-MeV (MOI 1) or with virulent G954-MeV (MOI 0.1). Autophagy was monitored by the numeration of GFP+ autophagosomes 24 h post infection in infected cells detected by a staining for the viral nucleoprotein N (MeV-N). Representative profiles for each condition are shown and are accompanied by a graph representing the number of GFP+ vesicles per cell profile ( = GFP+ vesicles per one nucleus). For syncytia, the number of dots was reported to the number of nuclei. Error bars, mean ± SD of three independent experiments for no infection and G954-MeV and one experiment for Ed-MeV. Student's t test; #p>0.05.
(TIF)

Figure S10 **Expression of CD150 on GFP-LC3 HeLa cells.** (A) GFP-LC3 Hela cells were transfected with a vector encoding for the expression of human CD150. 24 h post transfection, CD150 cell surface expression was monitored by FACS. (B) GFP-LC3 Hela cells were transduced with concentrated viral particles (peGAET-cd150-ires-puro plasmid) and CD150 was analysed by FACS on stably expressing CD150+ cells.
(TIF)

Figure S11 **G954-MeV-induced autophagy is ATG5 dependent.** GFP-LC3-HeLa cells were treated with the indicated siRNA for 24 h and transfected with a vector encoding for CD150. Cells were then infected with G954-MeV at MOI 0.1 (24 h) and autophagy was monitored by the numeration of GFP+ autophagosomes. Representative profiles are shown and are accompanied by a graph representing the number of GFP+ vesicles per cell profile ( = GFP+ vesicles per one nucleus). For syncytia, the number of dots was reported to the number of nuclei. Error bars, mean ± SD of three independent experiments. Student's t test; ***p<0.005.
(TIF)

Figure S12 **The second autophagic wave induced by G954-MeV requires viral protein synthesis.** CD150-transfected GFP-LC3-HeLa cells were infected or not with G954-MeV (MOI 0.1) or treated with 125 nM Rapa and treated or not with 0.5 µg/ml CHX. A staining against the MeV nucleoprotein N indicates infected cells. Representative profiles are shown and are accompanied by a graph representing the number of GFP+ vesicles per cell profile ( = GFP+ vesicles per one nucleus). For syncytia, the number of dots was reported to the number of nuclei. Error bars, mean ± SD of three independent experiments. Student's t test; ***p<0.005; **p<0.01; *p<0.05; #p>0.05.
(TIF)
