# Supplementary figures and images for: Sustained Autophagy Contributes to Measles Virus Infectivity
Source: PLoS Pathog. 2013 Sep 26;9(9):e1003599. doi: 10.1371/journal.ppat.1003599 (PMC3784470; doi:10.1371/journal.ppat.1003599)

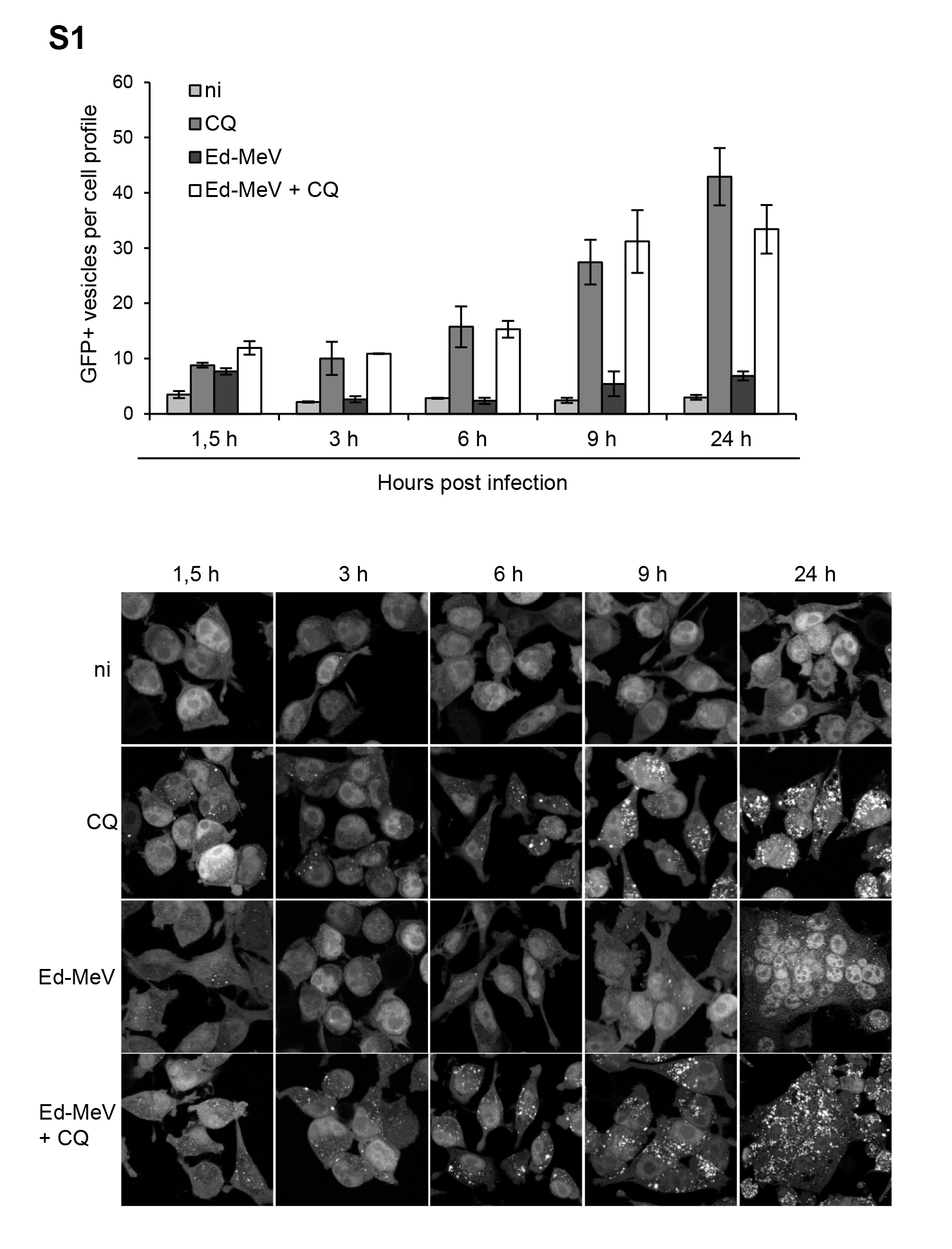

Supplement: Figure S1 — Effect of chloroquine during Ed-MeV infection. GFP-LC3 HeLa cells were infected with Ed-MeV at MOI 1 and/or treated with 75 µm Chloroquine (CQ). Autophagy was monitored by the numeration of GFP+ autophagosomes at the indicated period of time post infection. CQ was maintained all along during the culture. Representative profiles are shown and are accompanied by a graph representing the number of GFP+ vesicles per cell profile. Error bars, mean ± MD of two independent experiments. (TIF) [file ppat.1003599.s001.tif]

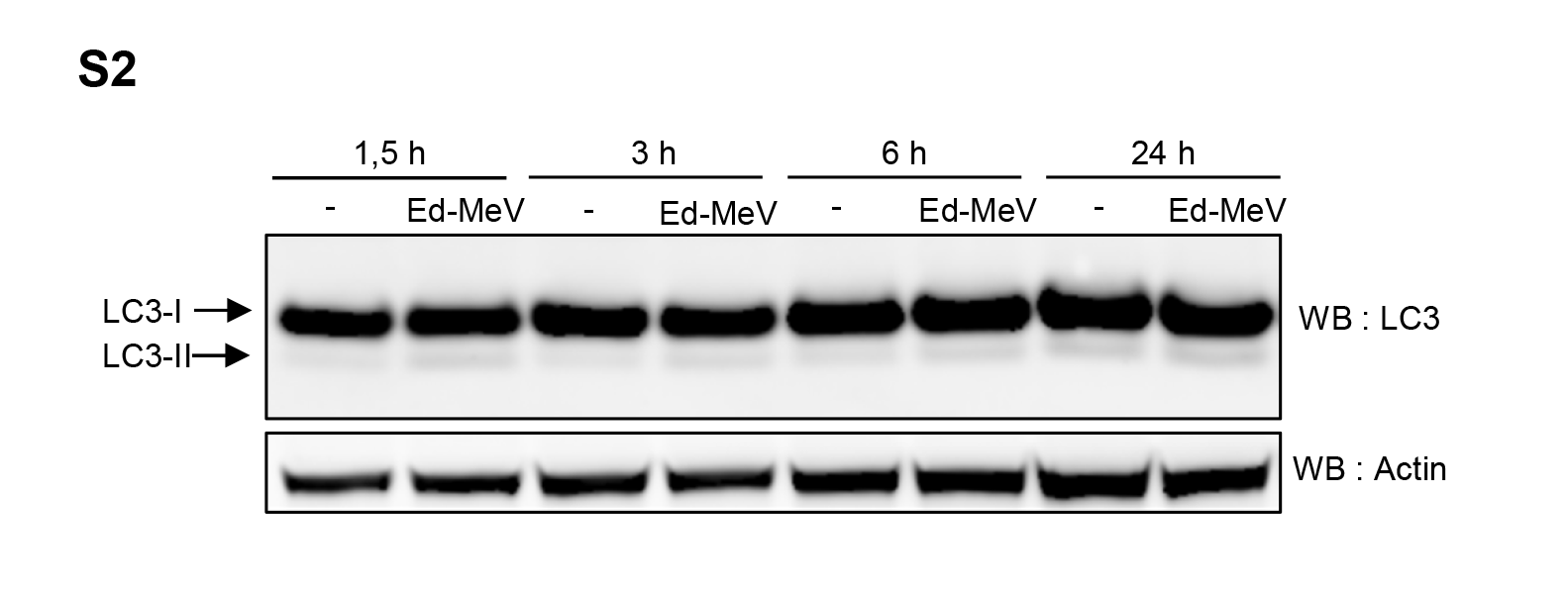

Supplement: Figure S2 — LC3-II expression during Ed-MeV infection. GFP-LC3-HeLa cells were infected with Ed-MeV (MOI 1). At the indicated time points post infection, autophagy was assessed by measuring LC3 conversion by western blot. (TIF) [file ppat.1003599.s002.tif]

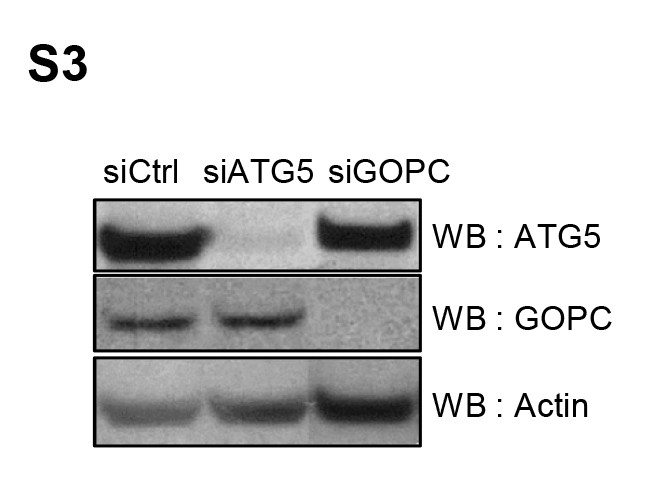

Supplement: Figure S3 — Efficiency of siRNA. HeLa cells were treated with the indicated siRNA. 48 h later, the expression of the respective proteins were assessed by western blot. (TIF) [file ppat.1003599.s003.tif]

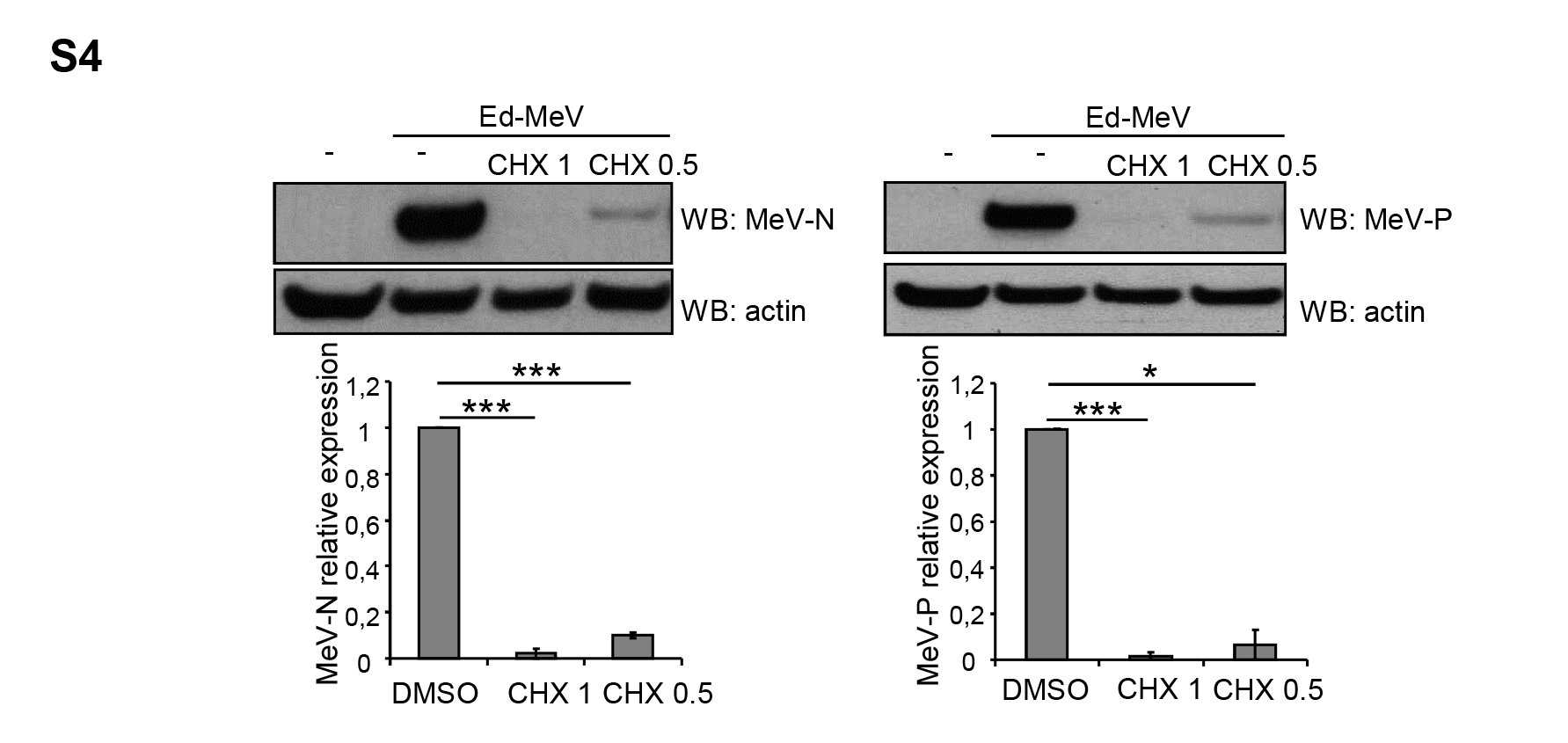

Supplement: Figure S4 — Efficiency of cycloheximide treatment. GFP-LC3 HeLa cells were infected with attenuated Ed-MeV (MOI 3) in the presence of 1 µg/ml or 0.5 µg/ml cycloheximide (CHX). 24 h post-infection, cells were lysed and anti-N and anti-P western blot were performed to reveal MeV-N and MeV-P, respectively. Representative results are shown and are accompanied by a graph representing the intensity of MeV-N or MeV-P expression over cellular actin, and normalized to the control condition (DMSO treatment). Error bars, mean ± SD of two independent experiments. Student's t test, ***p<0.005; *p<0.05. (TIF) [file ppat.1003599.s004.tif]

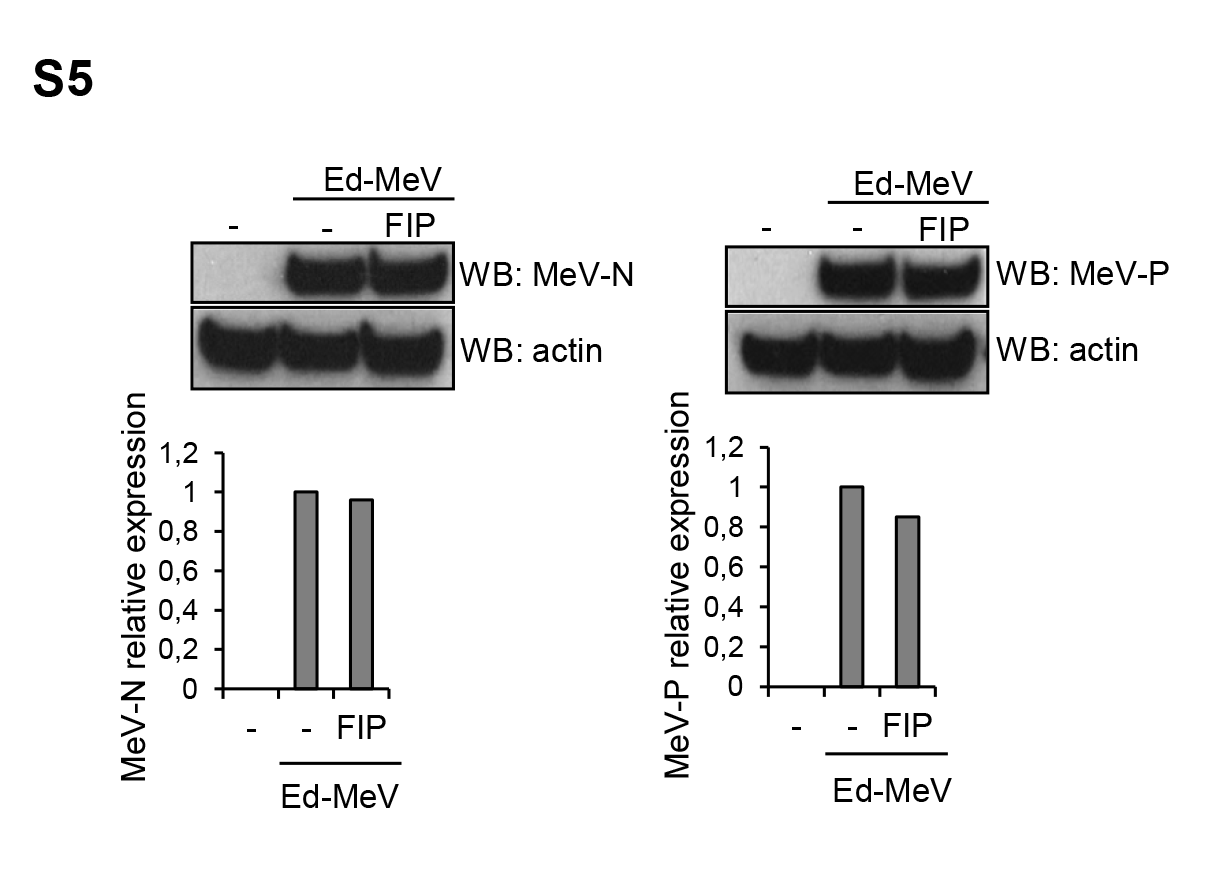

Supplement: Figure S5 — FIP treatment does not affect MeV viral protein synthesis. HeLa cells were infected or not with Ed-MeV (MOI 3) and treated or not with the FIP peptide (10 µg/mL). 24 h post-infection, cells were lysed and anti-N and anti-P western blot were performed to reveal MeV-N and MeV-P, respectively. Representative results are shown and are accompanied by a graph representing the intensity of MeV-N or MeV-P expression over cellular actin normalized to the control. (TIF) [file ppat.1003599.s005.tif]

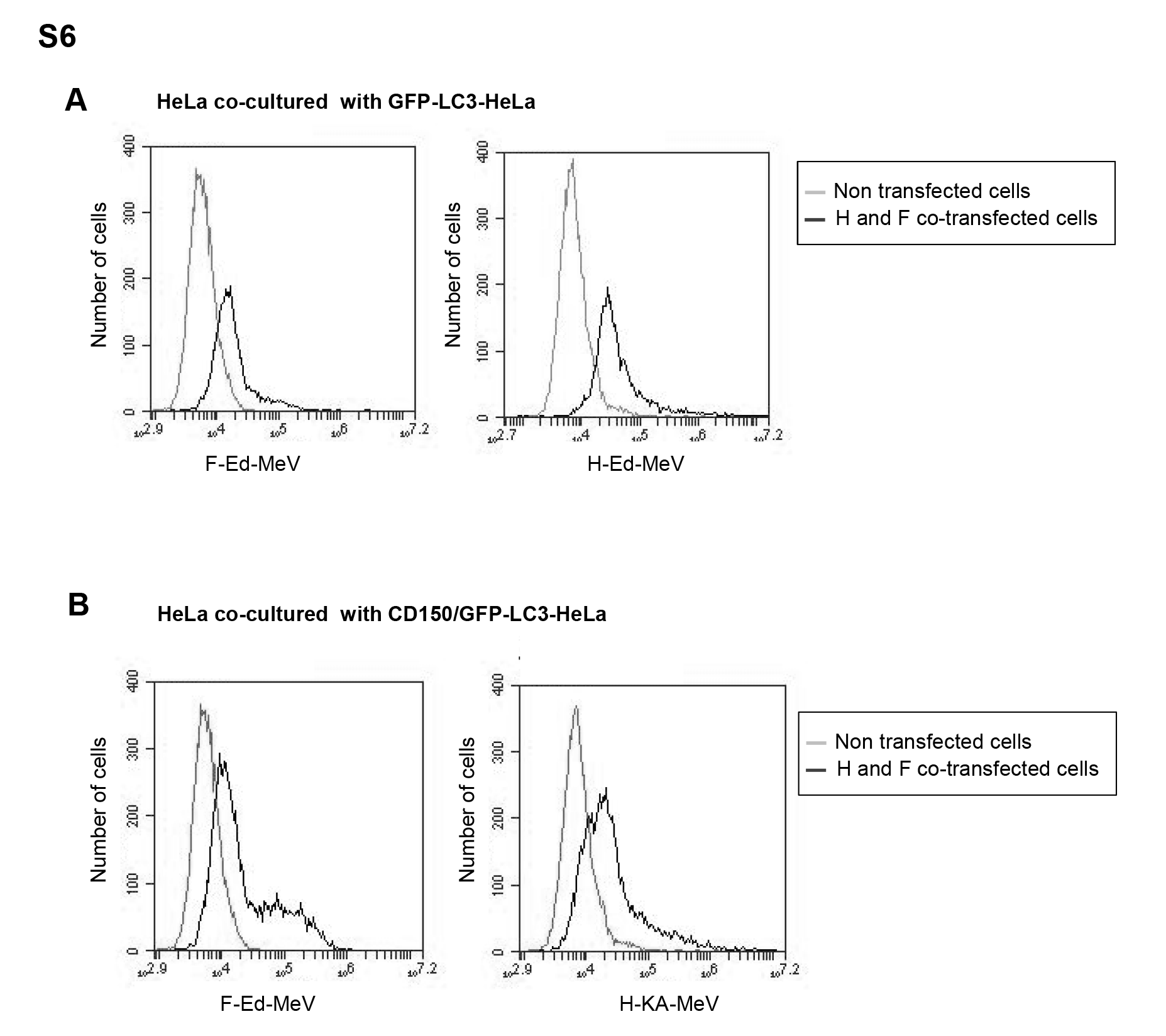

Supplement: Figure S6 — Expression of MeV-H and MeV-F on co-transfected cells. (A) HeLa cells were co-transfected with a vector encoding for the H protein of Ed-MeV (A) or the H protein of KA-MeV (B), and one encoding for the F protein (A and B). 24 h post transfection, expression of MeV-H and MeV-F was measured by FACS analysis. (TIF) [file ppat.1003599.s006.tif]

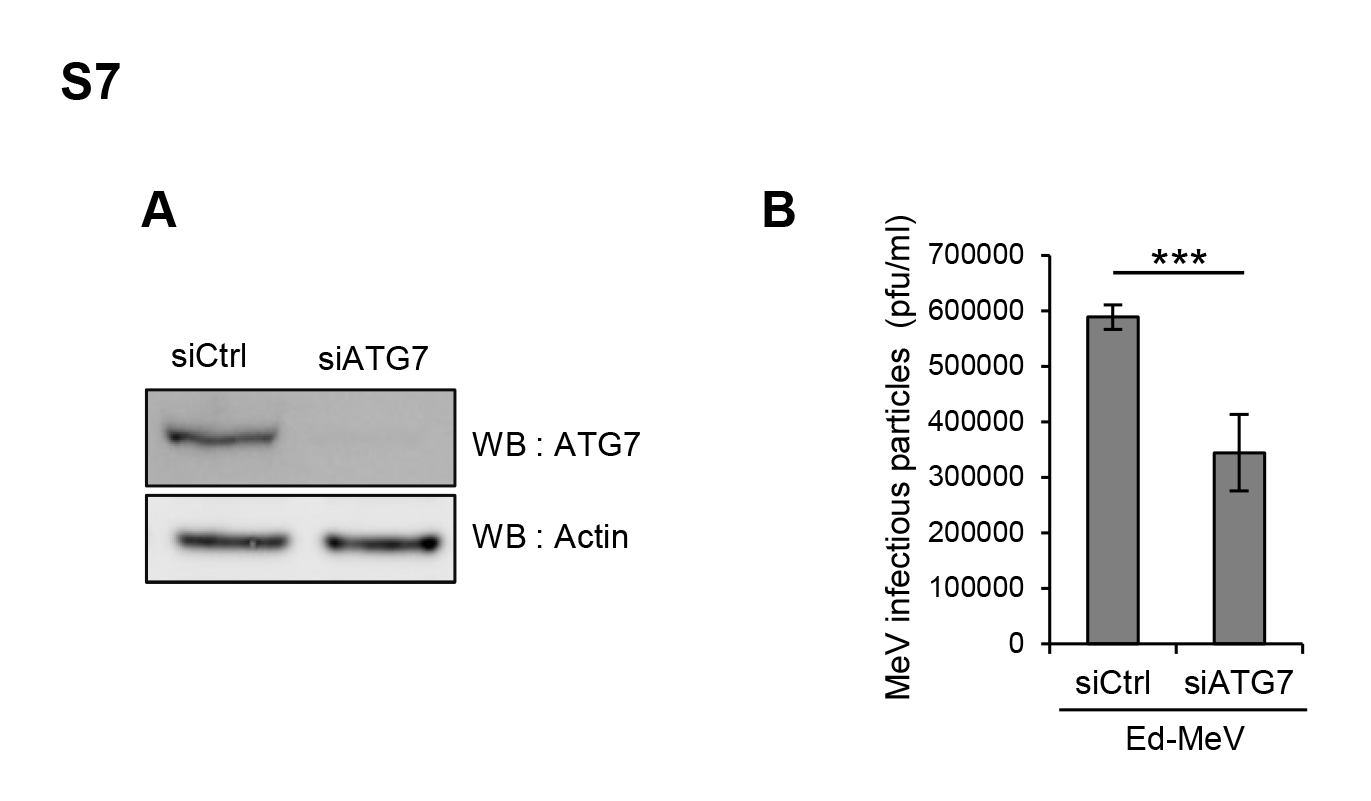

Supplement: Figure S7 — Ed-MeV particle formation is reduced in si ATG7 -treated cells. (A) the efficiency of siATG7 treatment is shown by western blot, for one representative experiment. (B) HeLa cells were treated with the indicated siRNA for 48 h and infected with Ed-MeV (MOI = 2). Two days post infection viral particles were titrated by plaque assays. Error bars, mean ± SD of six independent experiments. Student's t test; ***p<0.005. (TIF) [file ppat.1003599.s007.tif]

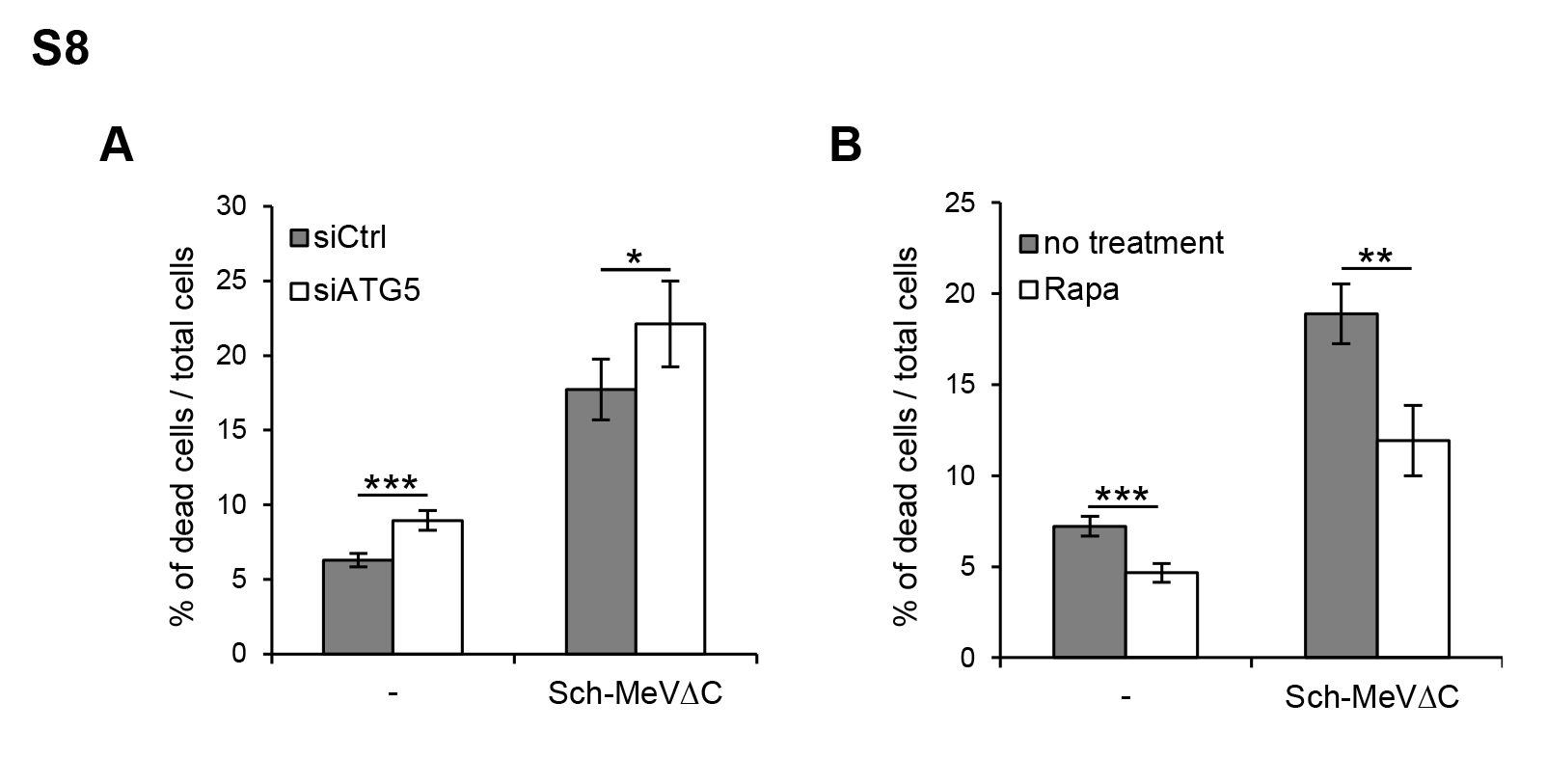

Supplement: Figure S8 — Autophagy protects Sch-MeVΔC infected cells from death. (A) HeLa cells were treated with the indicated siRNA for 48 h and infected or not with Sch-MeVΔC (MOI 0.1). (B) HeLa cells were treated or not with 250 nM rapamycin (Rapa) and infected or not with Sch-MeVΔC (MOI 1). (A, B) 48 h post infection, cell death was analysed by trypan blue exclusion test. Graphs represent the percentage of dead cells compared to the number of total cells. Error bars, mean ± SD of two independent experiments made in triplicate (A) and two independent experiments made in duplicate (B). (TIF) [file ppat.1003599.s008.tif]

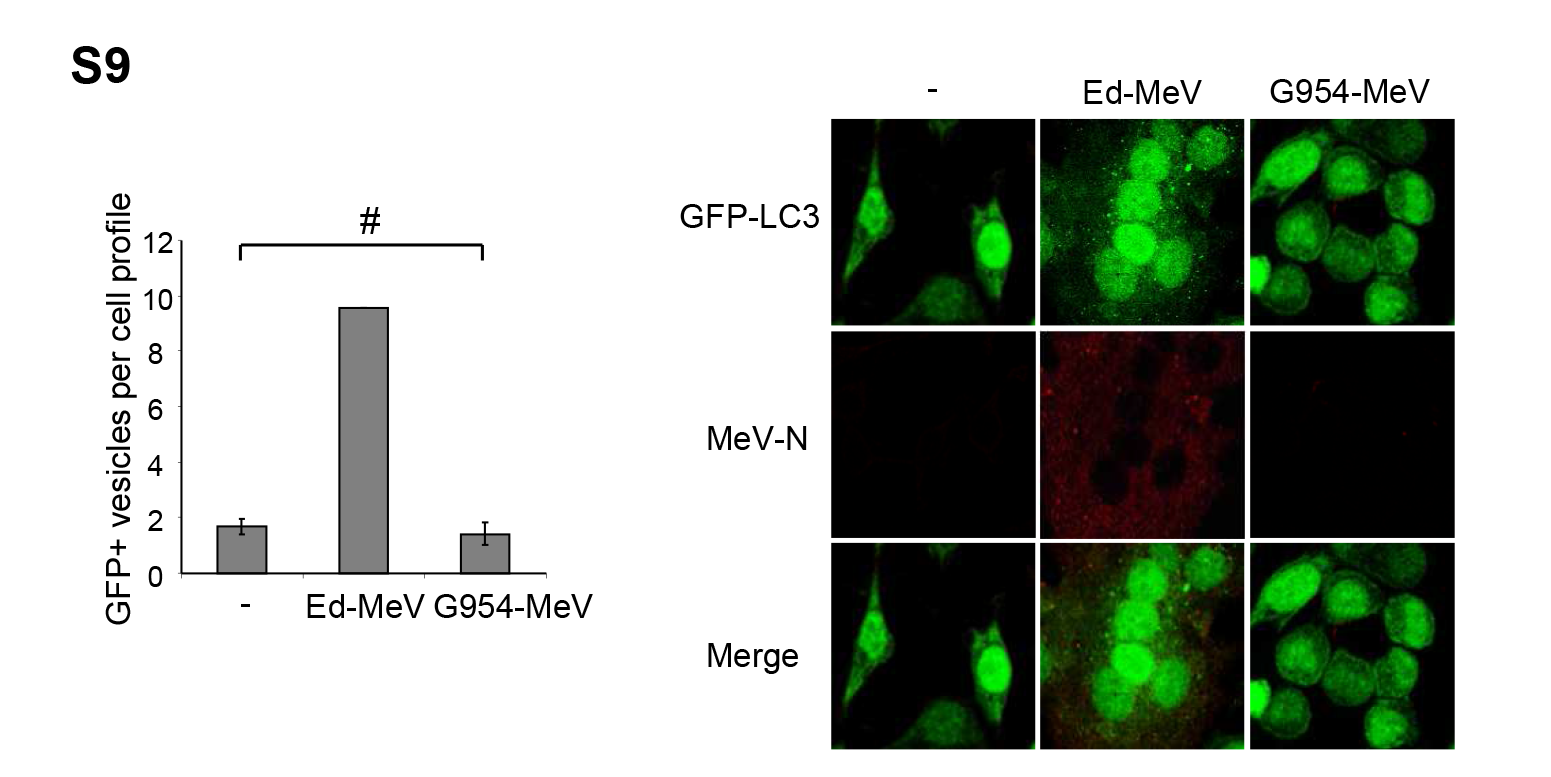

Supplement: Figure S9 — The virulent strain of measles virus does not induce autophagy in HeLa cells. GFP-LC3 HeLa cells were infected with attenuated Ed-MeV (MOI 1) or with virulent G954-MeV (MOI 0.1). Autophagy was monitored by the numeration of GFP+ autophagosomes 24 h post infection in infected cells detected by a staining for the viral nucleoprotein N (MeV-N). Representative profiles for each condition are shown and are accompanied by a graph representing the number of GFP+ vesicles per cell profile ( = GFP+ vesicles per one nucleus). For syncytia, the number of dots was reported to the number of nuclei. Error bars, mean ± SD of three independent experiments for no infection and G954-MeV and one experiment for Ed-MeV. Student's t test; #p>0.05. (TIF) [file ppat.1003599.s009.tif]

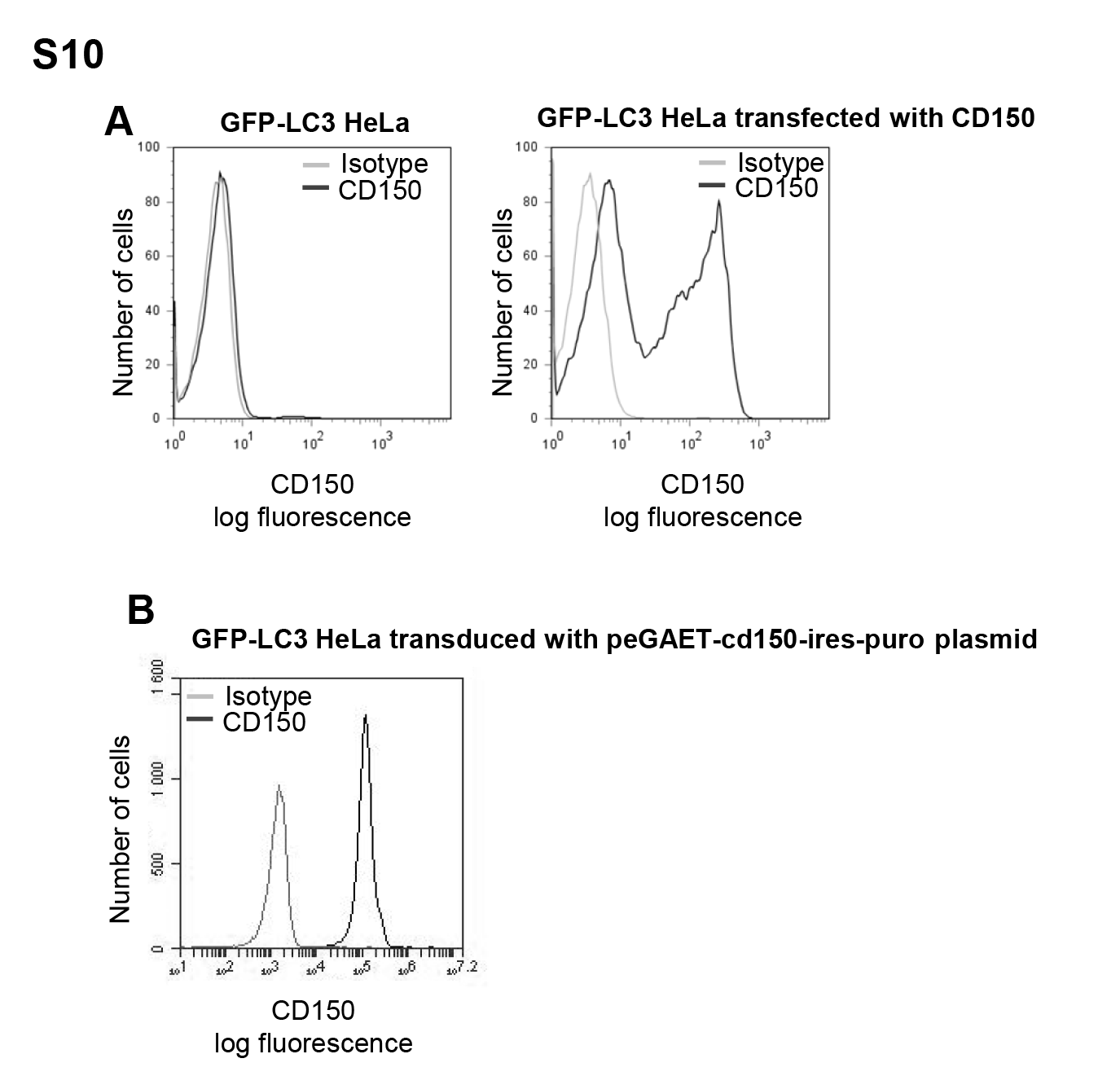

Supplement: Figure S10 — Expression of CD150 on GFP-LC3 HeLa cells. (A) GFP-LC3 Hela cells were transfected with a vector encoding for the expression of human CD150. 24 h post transfection, CD150 cell surface expression was monitored by FACS. (B) GFP-LC3 Hela cells were transduced with concentrated viral particles (peGAET-cd150-ires-puro plasmid) and CD150 was analysed by FACS on stably expressing CD150+ cells. (TIF) [file ppat.1003599.s010.tif]

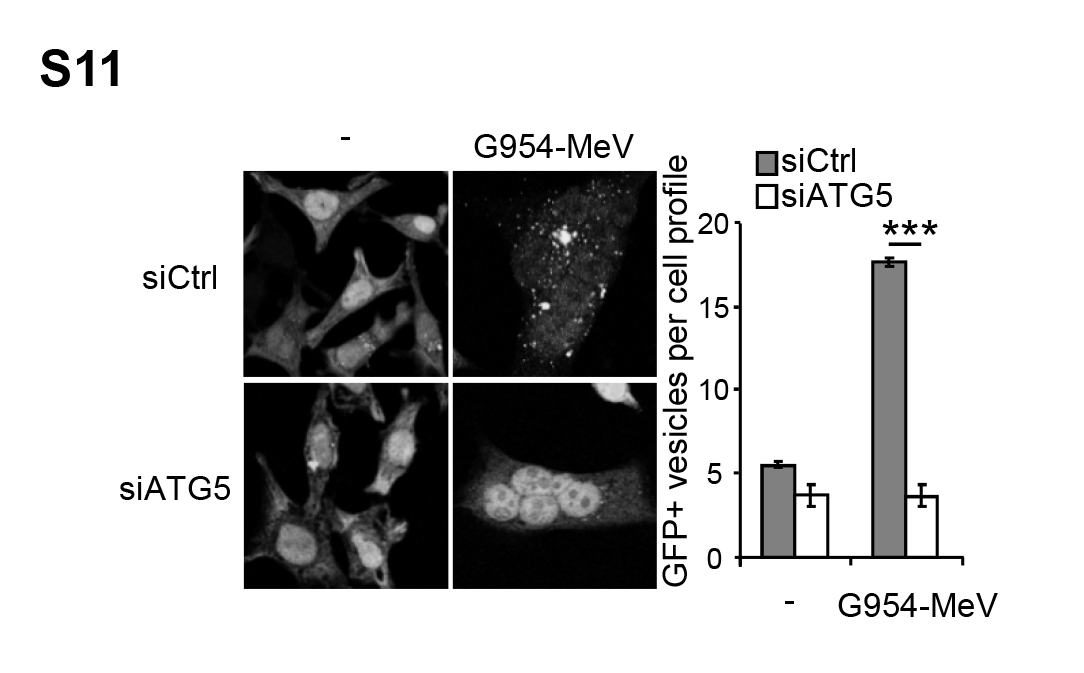

Supplement: Figure S11 — G954-MeV-induced autophagy is ATG5 dependent. GFP-LC3-HeLa cells were treated with the indicated siRNA for 24 h and transfected with a vector encoding for CD150. Cells were then infected with G954-MeV at MOI 0.1 (24 h) and autophagy was monitored by the numeration of GFP+ autophagosomes. Representative profiles are shown and are accompanied by a graph representing the number of GFP+ vesicles per cell profile ( = GFP+ vesicles per one nucleus). For syncytia, the number of dots was reported to the number of nuclei. Error bars, mean ± SD of three independent experiments. Student's t test; ***p<0.005. (TIF) [file ppat.1003599.s011.tif]

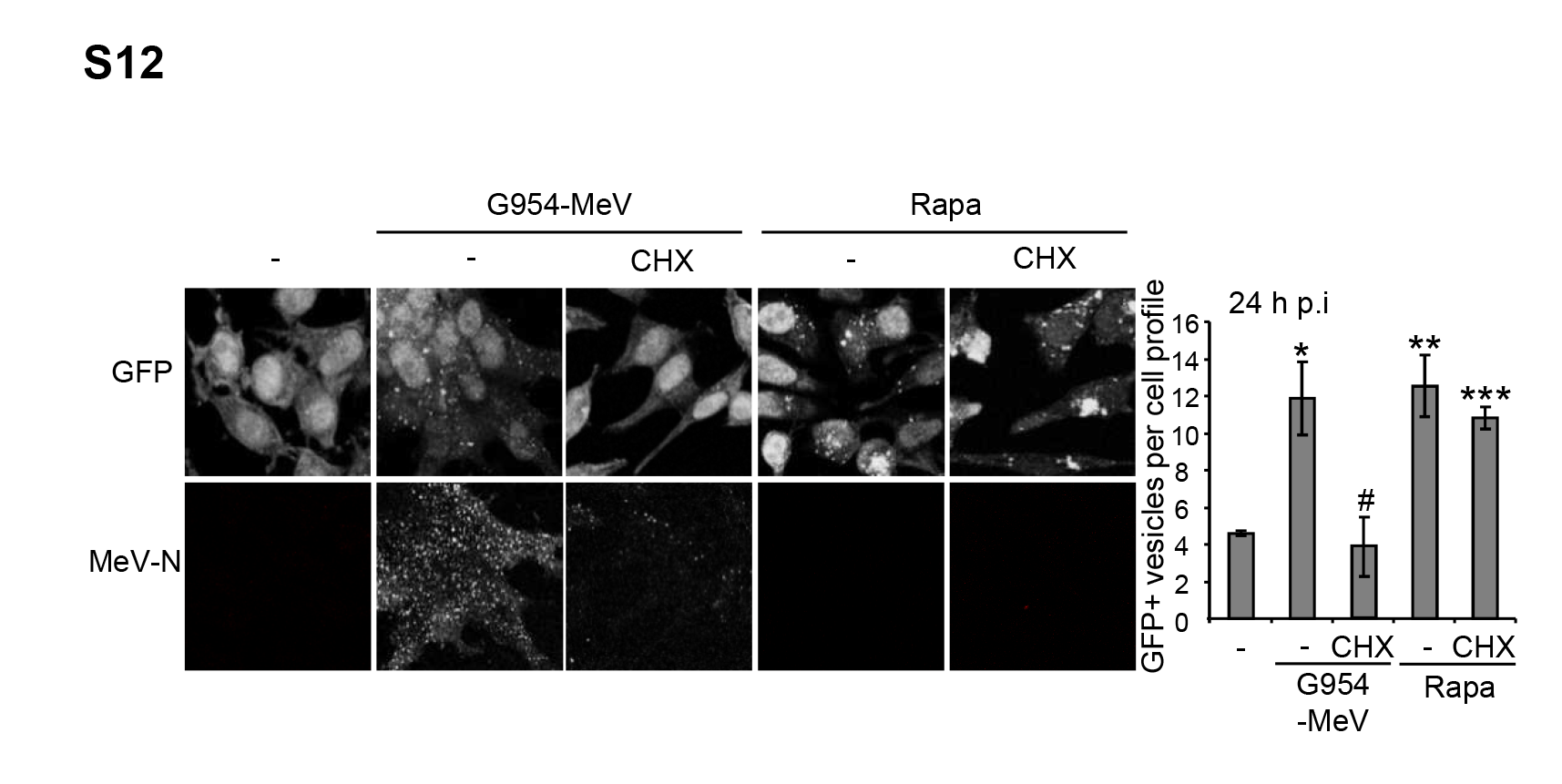

Supplement: Figure S12 — The second autophagic wave induced by G954-MeV requires viral protein synthesis. CD150-transfected GFP-LC3-HeLa cells were infected or not with G954-MeV (MOI 0.1) or treated with 125 nM Rapa and treated or not with 0.5 µg/ml CHX. A staining against the MeV nucleoprotein N indicates infected cells. Representative profiles are shown and are accompanied by a graph representing the number of GFP+ vesicles per cell profile ( = GFP+ vesicles per one nucleus). For syncytia, the number of dots was reported to the number of nuclei. Error bars, mean ± SD of three independent experiments. Student's t test; ***p<0.005; **p<0.01; *p<0.05; #p>0.05. (TIF) [file ppat.1003599.s012.tif]
